# Xylose Metabolism Perturbation in *Yarrowia lipolytica* for Efficient Succinic Acid Bioproduction from Lignocellulosic Biomass

**DOI:** 10.1002/advs.202507999

**Published:** 2025-08-19

**Authors:** Yutao Zhong, Changyu Shang, Jinhong Gu, Huilin Tao, Xuemei Lu, Jin Hou, Zhiyong Cui, Qingsheng Qi

**Affiliations:** ^1^ State Key Laboratory of Microbial Technology Shandong University Qingdao 266237 P. R. China

**Keywords:** fatty acid metabolism, lignocellulosic biomass, succinic acid biosynthesis, xylose catabolism, *Yarrowia lipolytica*

## Abstract

Lignocellulosic biomass is a sustainable feedstock for biorefineries, but inefficient xylose utilization limits microbial bioproduction. Here, the oleaginous yeast *Yarrowia lipolytica* was engineered to produce succinic acid (SA) from xylose by resolving metabolic and regulatory conflicts. Initial overexpression of xylose catabolic genes (*XR*, *XDH*, *XK*) in an SA‐hyperproducing strain did not activate xylose utilization, indicating underlying cryptic constraints. Adaptive evolution identified critical mutations (*Snf1^R78W^*, *Scp1^delGTC^*) that globally downregulated downstream pathways, including glycolysis and β‐oxidation, restoring growth using xylose but reducing SA production. To overcome this trade‐off, a random expression library strategy incorporating multi‐copy amplification of *XR*, *XDH*, and *XK* genes via nonhomologous end joining (NHEJ) was employed. This approach significantly enhanced xylose utilization and SA production, achieving 83.78 g L^−1^ SA from corn stover hydrolysate at pH 3.5 (yield: 0.66 g g^−1^ mixed sugars; productivity: 1.21 g L^−1^ h^−1^). Mechanistic studies revealed that fatty acid metabolism drives a futile cycle converting cytosolic NADPH to mitochondrial NADH, essential for SA biosynthesis via the reductive TCA pathway. This cycle competitively inhibits xylose catabolism unless pathway genes are amplified to balance cofactor demand. This work highlights the importance of fatty acid metabolism in *Y. lipolytica* for SA biosynthesis, cofactor rebalancing, and pathway cross‐talks.

## Graphical abstract

This study engineers *Yarrowia lipolytica* to produce succinic acid (SA) from lignocellulosic biomass. Adaptive evolution and multi‐omics analysis identify regulators that alleviate xylose metabolic disorder. Multi‐copy integration of xylose metabolic genes resolves redox imbalances from a futile fatty acid cycle, producing high‐titer SA from corn stover hydrolysate at low pH. This establishes a cofactor‐driven lignocellulosic biorefinery platform.

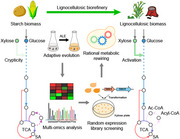

## 1 Introduction

Lignocellulosic biomass has emerged as a carbon‐neutral alternative to fossil resources for the synthesis of biofuels and biochemicals, with xylose being the second most abundant fermentable sugar after glucose, comprising 30%–40% of total carbohydrates.^[^ ^1^, ^2^, ^3^ ^]^ However, the microbial conversion of xylose in industrial strains remains inefficient due to the absence of native high‐flux xylose catabolic pathways and regulatory interference with central carbon metabolism.^[^ ^4^, ^5^, ^6^ ^]^ While metabolic engineering has successfully enabled xylose fermentation in conventional ethanol producers such as *Saccharomyces cerevisiae* and *Zymomonas mobilis*, their narrow product spectrum and reliance on glucose‐centric feedstocks highlight the necessity for more versatile microbial platforms that can robustly co‐utilize mixed sugars.^[^ ^7^, ^8^ ^]^

The oleaginous yeast *Yarrowia lipolytica* has emerged as a robust microbial platform for the biosynthesis of organic acids, attributed to its high tricarboxylic acid (TCA) cycle flux and tolerance to acidic conditions.^[^ ^9^, ^10^, ^11^ ^]^ This yeast strain has been successfully engineered for low‐pH succinic acid (SA) production—a pivotal C4 platform chemical—eliminating the need for neutralization steps required in bacterial production systems.^[^ ^12^, ^13^, ^14^, ^15^, ^16^, ^17^, ^18^ ^]^ In our previous work, we engineered a mitochondrial reductive TCA (rTCA) pathway in *Y. lipolytica* to overcome cytosolic NADH limitations, achieving 111.7 g L^−1^ SA with a yield of 0.79 g g^−1^ glucose without pH control.^[^ ^19^ ^]^ However, the inability of these engineered strains to co‐utilize xylose, a major component of lignocellulosic hydrolysates, limits their economic viability in biorefineries.

Efficient xylose catabolism is crucial for the economic bioconversion of lignocellulosic biomass into value‐added products.^[^ ^20^ ^]^ While the regulatory networks of xylose metabolism have been thoroughly characterized in *S. cerevisiae*, enabling successful engineering strategies to overcome carbon catabolite repression and optimize utilization efficiency,^[^ ^21^, ^22^ ^]^ the corresponding mechanisms in non‐conventional hosts like *Y. lipolytica* remain poorly understood.^[^ ^23^ ^]^ In yeasts, xylose assimilation relies on the oxidoreductase pathway: xylose reductase (XR) reduces xylose to xylitol using NADPH, xylitol dehydrogenase (XDH) oxidizes xylitol to xylulose with NAD⁺, and xylulokinase (XK) phosphorylates xylulose to Xu5P for entry into central metabolism.^[^ ^24^, ^25^ ^]^ Although *Y. lipolytica* possesses intact pathway genes (*XR*, *XDH*, and *XK*), native strains are unable to metabolize xylose.^[^ ^26^ ^]^ Cryptic catabolism can be activated by overexpressing these genes with strong promoters, but this approach alone is insufficient to achieve robust xylose utilization.^[^ ^27^, ^28^, ^29^, ^30^ ^]^

A critical bottleneck in the xylose assimilation pathway is the inherent redox imbalance: XR consumes NADPH, while XDH generates NADH, leading to competing cofactor demands that disrupt metabolic flux.^[^ ^22^ ^]^ Cofactor engineering strategies have significantly improved xylose utilization in *S. cerevisiae*. These strategies include altering the cofactor preference of XR to NADH to improve ethanol production,^[^ ^31^ ^]^ enhancing cofactor regeneration to boost 2,3‐butanediol production,^[^ ^32^ ^]^ and using promoter engineering to optimize the NADH/NADPH balance in xylose metabolism.^[^ ^33^ ^]^ Despite these advancements, their application in *Y. lipolytica* for the production of high‐value products remains largely unrealized. For example, when *Y. lipolytica* was engineered for lipid overproduction (73% cell dry weight) using a “push‐pull‐block” strategy, it completely lost the ability to utilize xylose, despite the integration of heterologous xylose pathways.^[^ ^34^ ^]^ Although adaptive evolution partially restored growth on xylose, the mechanistic basis—potentially involving cofactor rebalancing or pathway crosstalk—remains unresolved.^[^ ^34^ ^]^ These observations reveal a systemic incompatibility between xylose catabolism and NADPH‐dependent pathways, such as lipid biosynthesis, underscoring the need for cofactor‐aware metabolic designs.

To overcome metabolic regulatory constraints and enable lignocellulosic SA production in *Y. lipolytica*, we systematically perturbed xylose metabolism through adaptive laboratory evolution (ALE) and multi‐copy expression screening. ALE combined with multi‐omics analysis revealed interference patterns between xylose metabolism and SA synthesis. By screening a random expression library for xylose‐growth‐coupled variants, we identified clones with amplified gene copy numbers of *XR* (3.36 copies per cell), *XDH* (2.40 copies per cell), and *XK* (4.32 copies per cell) as essential for functional xylose catabolism. The optimized strain achieved SA production of 85.48 g L^−1^ from xylose and 83.78 g L^−1^ SA from corn stover hydrolysate at low pH, demonstrating its industrial feasibility. Further mechanistic investigations revealed that fatty acid metabolism is indispensable for SA biosynthesis and influences xylose catabolism through cytosolic NADPH and mitochondrial NADH cycling. This study advances the fundamental understanding of xylose metabolism in non‐model yeasts and establishes *Y. lipolytica* as an industrial platform for lignocellulosic biorefineries.

## 2 Results

### 2.1 Overexpression of Xylose Metabolism Genes Cannot Enable SA‐Producing *Y. lipolytica* to Utilize Xylose

**Figure 1:**
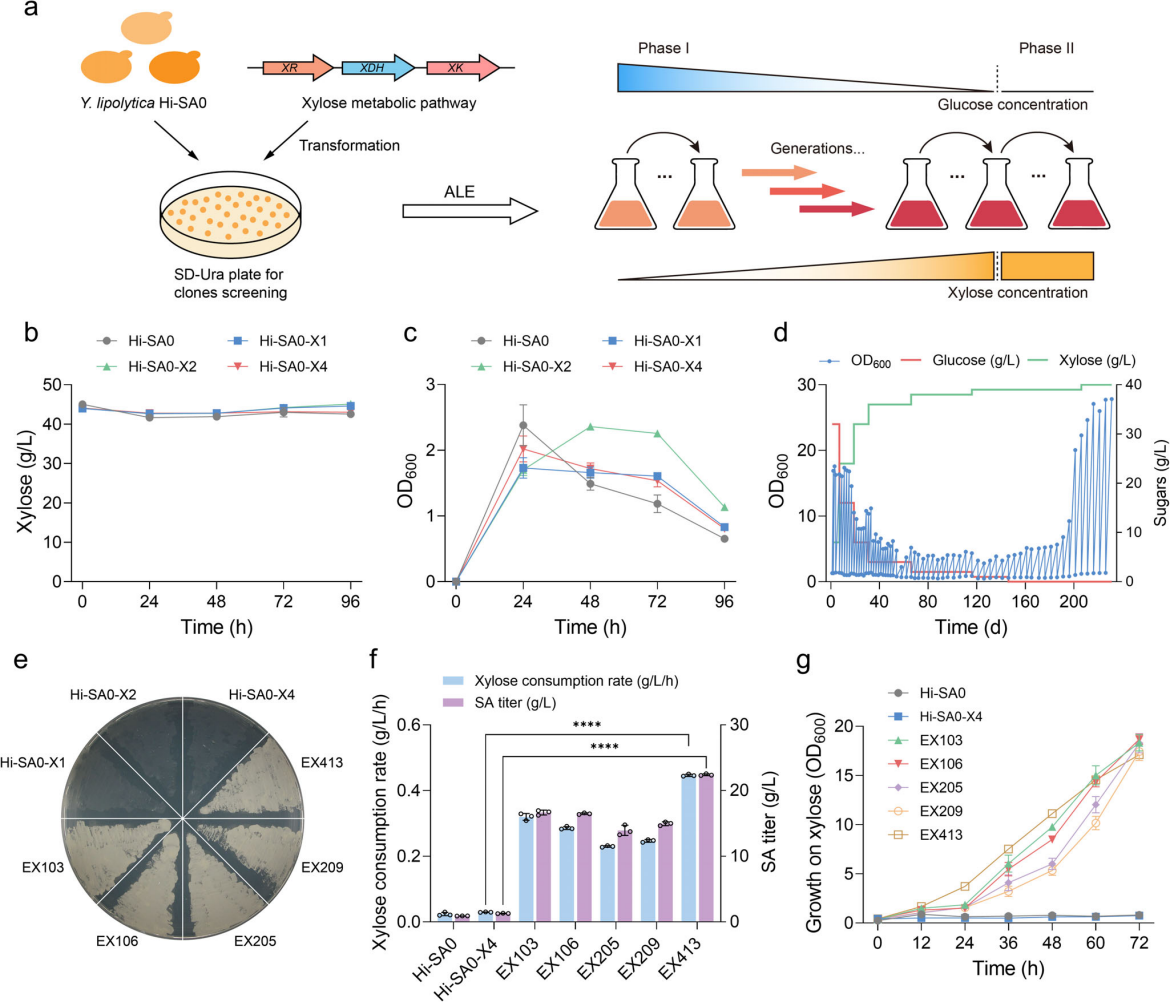
Adaptive laboratory evolution of SA‐producing *Y. lipolytica* to restore xylose utilization and growth on xylose. a) Schematic of xylose metabolic pathway engineering and the ALE process. Comparison of b) xylose consumption and c) cell growth of Hi‐SA0‐X1, Hi‐SA0‐X2, and Hi‐SA0‐X4 in YPX medium. d) Time‐course of cell growth (OD_600_) and sugars concentrations during the ALE of Hi‐SA0‐X4. e) Growth recovery of evolved strains on YPX plates. Comparison of f) xylose consumption rate, SA titer, and g) cell growth between unevolved and evolved strains in YPX medium. Error bars represent mean ± s.d. (*n* = 3 biologically independent samples). Statistical analysis was performed using a two‐tailed Student's *t*‐test (****p < 0.0001).

To enhance xylose utilization, key enzymes involved in the native xylose metabolic pathway (including XR, XDH, and XK) were overexpressed in the engineered *Y. lipolytica* strain Hi‐SA0, which contains a mitochondrially localized rTCA cycle (**Figure** **1**a). Transformants were selected on uracil dropout plates following random NHEJ‐mediated integration of the expression cassette containing the *URA3* selectable marker. However, following extensive screening of over 100 transformants, the selected strains Hi‐SA0‐X1, Hi‐SA0‐X2, and Hi‐SA0‐X4 remained unable to efficiently utilize xylose as the sole carbon source for growth and SA production, accompanied by the accumulation of ≈0.6 g L^−1^ xylitol (Figure 1b,c; Figure S1, Supporting Information). This result was not consistent with a previous report that showed robust growth and SA production in succinate dehydrogenase (SDH)‐deficient *Y. lipolytica* strains with overexpressed endogenous xylose metabolic pathways when grown in xylose medium.^[^ ^35^ ^]^ Unlike the oxidative TCA pathway in the aforementioned strain, the enhanced rTCA pathway in Hi‐SA0 may interfere with xylose catabolism, potentially impairing xylose utilization. Furthermore, our attempts to overexpress xylose‐specific transporters and the non‐oxidative pentose phosphate pathway (PPP) in strain Hi‐SA0‐X4 also failed to enable the strain to utilize xylose (data not shown).

### 2.2 Restoring Xylose Utilization of SA‐Producing *Y. lipolytica* through Adaptive Laboratory Evolution (ALE)

An ALE approach was employed to restore the xylose utilization capability of the *Y. lipolytica* strain Hi‐SA0‐X4. Serial transfer experiments were conducted over ≈200 generations in glucose‐xylose medium. The early generations demonstrated rapid growth because of the abundance of glucose, but gradually adapted to low‐glucose and high‐xylose conditions (Figure 1d). The final evolved strains were capable of efficient growth in xylose medium. Following random screening of ≈30 clones from each evolved population for SA production using xylose as the sole carbon source, strains EX103, EX106, EX205, EX209, and EX413 were isolated from evolutionary endpoints. These evolved strains demonstrated visible growth on YPX plates, whereas unevolved strains (e.g., Hi‐SA0‐X4) showed no growth (Figure 1e). These evolved strains exhibited a significant increase in xylose consumption rate and SA titer, indicating effective metabolic rewiring. Among them, strain EX413 achieved the highest SA titer of 22.4 g L^−1^ within 72 h and the fastest xylose consumption rate of 0.45 g L^−1^ h^−1^ (Figure 1f). Moreover, strain EX413 demonstrated significantly improved growth on xylose with a shorter lag phase compared to the other four evolved strains (Figure 1g).

### 2.3 Genetic and Metabolic Characteristics of Evolved Strains with Restored Xylose Metabolism

**Figure 2:**
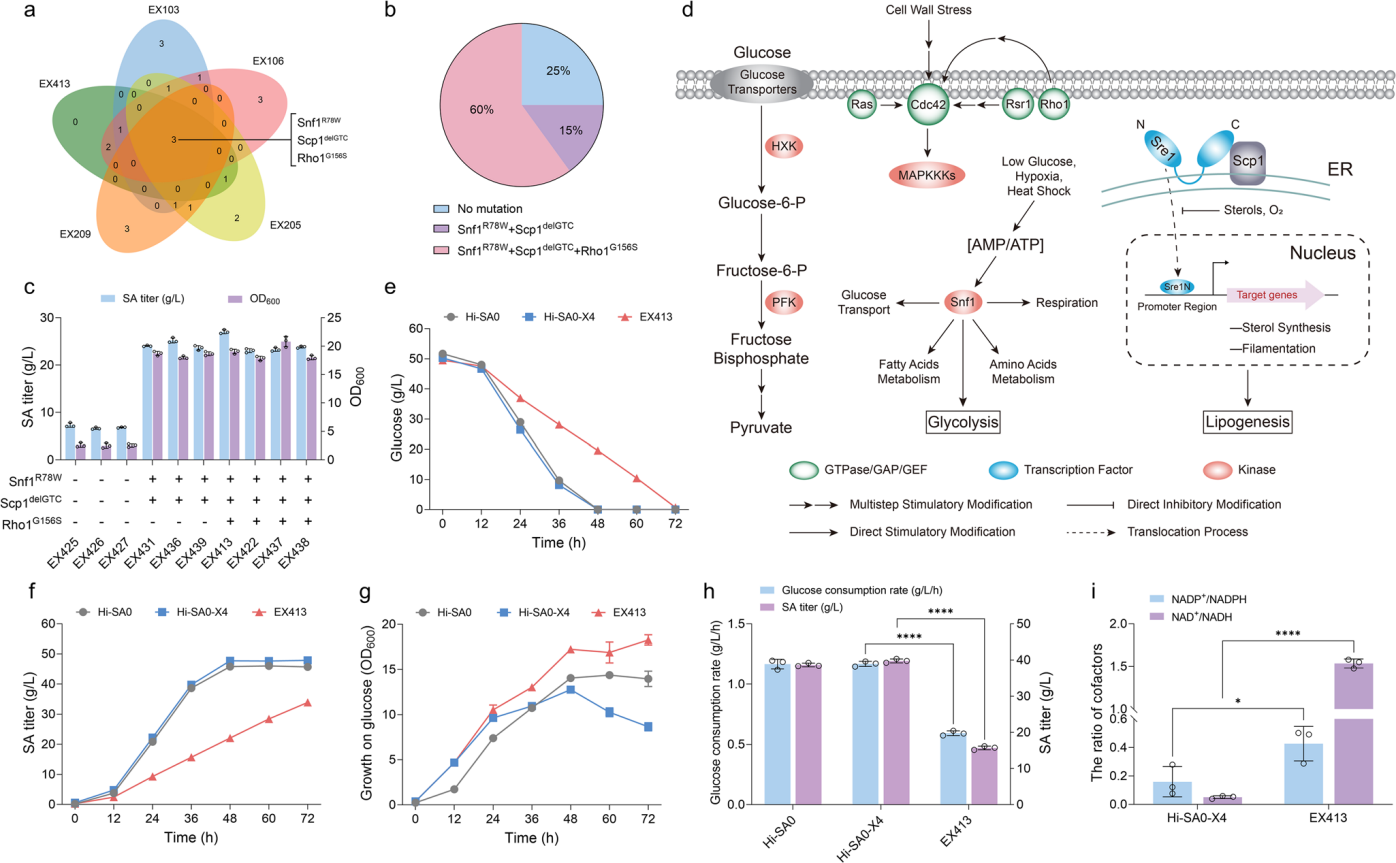
Genetic and metabolic characterization of evolved *Y. lipolytica* strains with restored growth on xylose. a) Venn diagram of mutations (SNVs and InDels) in five evolved strains. b) Distribution of mutation combinations in the evolved endpoint population. c) SA titer and cell growth (OD_600_) of strains with different mutations in YPX medium. d) Schematic illustration of the hypothetical signaling pathways involved in Snf1, Scp1, and Rho1. Rsr1: Ras‐related protein; Cdc42: Cell division control protein 42 homolog; Rho1: Ras homolog gene family; Sre1: Sterol regulatory element‐binding protein; Scp1: Sre1 cleavage‐activating protein; MAPKKKs: Mitogen‐activated protein kinase kinase kinases; AMP: Adenosine monophosphate; ATP: Adenosine triphosphate; HXK: Hexokinase; PFK: Phosphofructokinase; Snf1: Sucrose non‐fermenting 1 protein kinase; GTPase: Guanosine triphosphatase; GAP: GTPase‐activating protein; GEF: Guanine nucleotide exchange factor; ER: Endoplasmic reticulum; Glucose‐6‐P: Glucose‐6‐phosphate; Fructose‐6‐P: Fructose‐6‐phosphate. Comparison of e) glucose consumption, f) SA titer, and g) cell growth between unevolved strains and evolved strain EX413 in YPD medium. h) Glucose consumption rate and SA titer of unevolved strains and evolved strain EX413 within 36 h in YPD medium. i) The ratios of [NADP^+^]/[NADPH] and [NAD^+^]/[NADH] in Hi‐SA0‐X4 and EX413. Error bars represent mean ± s.d. (*n* = 3 biologically independent samples). Statistical analysis was performed using a two‐tailed Student's *t*‐test (*p < 0.05, ****p < 0.0001).

To investigate the potential mechanisms underlying the restored xylose utilization, genome resequencing was performed for five isolated clones from the evolved population (EX103, EX106, EX205, EX209, and EX413). A total of 22 single‐nucleotide variations (SNVs) and insertions/deletions (InDels) were identified in coding regions compared with the unevolved strain Hi‐SA0‐X4 (Table S1, Supporting Information). Notably, shared mutations Snf1^R78W^, Scp1^delGTC^, and Rho1^G156S^, were found in all five clones (**Figure** **2**a). These mutations were confirmed in these five evolved strains by PCR validation. Subsequent PCR screening of 20 single colonies from the endpoint population revealed a varying distribution of these mutations: 60% of the colonies harbored all three mutations, 15% had Snf1^R78W^ and Scp1^delGTC^ mutations, and 25% had none of these mutations (Figure 2b). Among these colonies, the strains without any mutations (EX425, EX426, and EX427) displayed poor growth and SA production in xylose medium (Figure 2c). The strains carrying Snf1^R78W^ and Scp1^delGTC^ mutations (EX431, EX436, and EX439) exhibited similar growth and SA production capabilities to those with all three mutations (EX422, EX437, and EX438), suggesting that Snf1^R78W^ and Scp1^delGTC^ are more important for enhancing xylose utilization within these mutations (Figure 2c).

Snf1, a serine/threonine protein kinase activated by low glucose levels, hypoxia, or heat shock, orchestrates metabolic shifts in yeast in response to stress by promoting respiration, regulating fatty acid and amino acid metabolism, and controlling glucose transport and glycolysis.^[^ ^36^, ^37^ ^]^ Scp1, in complex with Sre1, regulates fatty acid metabolism in response to sterol levels and oxygen availability.^[^ ^38^ ^]^ Upon activation, Sre1 is translocated to the nucleus to regulate genes involved in sterol synthesis and filamentation (Figure 2d; Table S2, Supporting Information). Both Snf1 and Scp1 are regulators of cellular metabolism, including glucose metabolism and fatty acid metabolism, while Rho1 is a small GTPase that regulates cell wall biosynthesis and maintenance.^[^ ^39^ ^]^ Because of the ineffective genetic manipulation of the strain Hi‐SA0‐X4, reverse engineering was performed in the wild‐type *Y. lipolytica* strain Po1fX, which expressed the xylose assimilation pathway, to validate the effects of the identified mutations on glucose and xylose metabolism. The strain Po1fX‐Snf1^R78W^ exhibited a 6.5% lower glucose consumption rate compared with the control strain Po1fX‐Snf1. The Scp1^delGTC^ mutation resulted in a 25.9% faster xylose consumption rate and improved growth on xylose, but a 7.1% slower glucose consumption rate compared with the control strain Po1fX‐Scp1 (Figure S2 and Table S3, Supporting Information). In contrast, the Rho1^G156S^ mutation had negligible effects on xylose or glucose metabolism and cell growth. These results suggest that the mutation Scp1^delGTC^ promotes xylose utilization, while Snf1^R78W^ inhibits glucose metabolism. Further transcriptome analysis of the three mutant strains in xylose medium revealed 66 shared differentially expressed genes (DEGs) (Figure S3a, Supporting Information). Among these, most genes involved in fatty acid metabolism were downregulated and primarily enriched according to Gene Ontology (GO) enrichment analysis (Figure S3b,c and Table S4, Supporting Information). The mutations identified in these regulatory factors suggest a global perturbation of cellular metabolism in *Y. lipolytica* cells, which adapts to xylose‐rich conditions by slowing central carbon metabolism and fatty acid metabolism.

Therefore, we evaluated the SA fermentation performance of strain EX413 in media containing glucose as a sole carbon source and mixed sugars (glucose and xylose). Although the evolved strain EX413 exhibited better growth compared with the control strain Hi‐SA0‐X4, its SA production and glucose consumption rate were significantly reduced by 60.6% and 49.3%, respectively (Figure 2e–h). During mixed sugar fermentation, strain EX413 demonstrated the ability to co‐ferment glucose and xylose; however, it still showed inefficient glucose metabolism (Figure S4, Supporting Information). We also examined intracellular redox levels and found that the [NAD^+^]/[NADH] ratio in strain EX413 was 29.44‐fold higher than that in strain Hi‐SA0‐X4, indicating that the metabolic pathways related to NADH generation may be weakened in the xylose growth‐restored strains (Figure 2i). These results show that the non‐rational evolution strategy could successfully induce mutations that enhanced the xylose utilization of *Y. lipolytica* but compromised its ability to efficiently metabolize glucose.

### 2.4 Transcriptional Profiling of the Evolved Strain EX413

Transcriptome analysis was employed to elucidate the mechanisms underlying the restored xylose utilization and inefficient glucose metabolism observed in the evolved strain EX413. DEGs were visualized through a volcano plot, with genes exhibiting a log_2_ fold change ≥ 1 in expression and a q‐value ≤ 0.05 defined as DEGs (Figure S5, Supporting Information). A comparison between the evolved strain EX413 and the parental strain Hi‐SA0‐X4 revealed a total of 890 DEGs, of which 391 were upregulated and 499 were downregulated (Table S5, Supporting Information).

**Figure 3:**
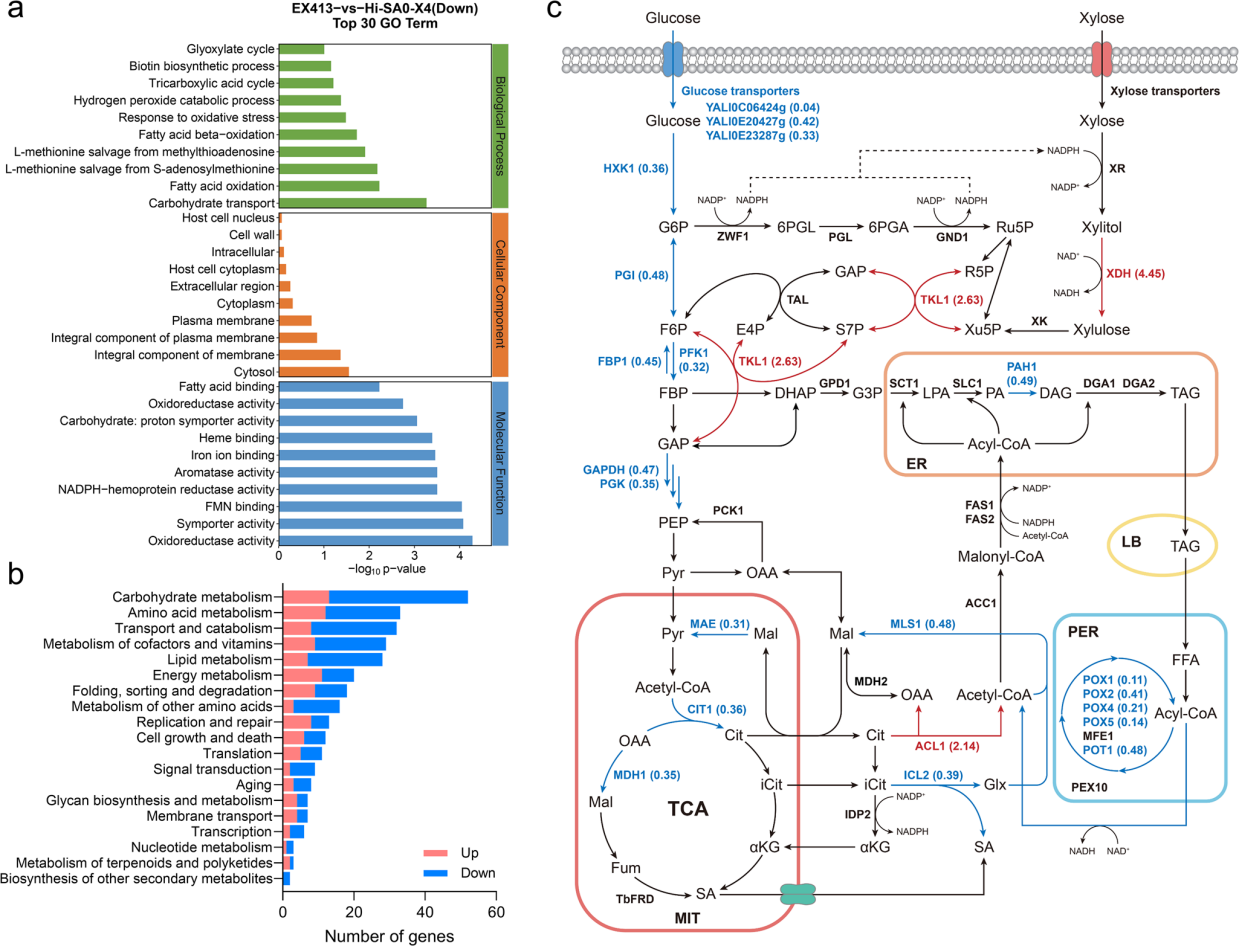
Transcriptome analysis of the metabolic perturbation in the evolved strain EX413. a) Top 30 downregulated GO terms of DEGs between the evolved strain EX413 and the unevolved strain Hi‐SA0‐X4. b) KEGG pathway enrichment analysis of upregulated and downregulated genes between EX413 and Hi‐SA0‐X4. c) Changes in transcriptional levels of genes involved in the central carbon metabolic pathway of the evolved strain EX413. G6P: Glucose‐6‐phosphate; F6P: Fructose‐6‐phosphate; FBP: Fructose‐1,6‐bisphosphate; GAP: Glyceraldehyde‐3‐phosphate; DHAP: Dihydroxyacetone phosphate; G3P: Glycerol‐3‐phosphate; PEP: Phosphoenolpyruvate; Pyr: Pyruvate; OAA: Oxaloacetate; Mal: Malate; Cit: Citrate; iCit: Isocitrate; αKG: Alpha‐ketoglutarate; Fum: Fumarate; LPA: Lysophosphatidic acid; PA: Phosphatidic acid; DAG: Diacylglycerol; TAG: Triacylglycerol; FFA: Free fatty acids; Glx: Glyoxylate; 6PGL: 6‐Phosphogluconolactone; 6PGA: 6‐Phosphogluconate; Ru5P: Ribulose‐5‐phosphate; R5P: Ribose‐5‐phosphate; Xu5P: Xylulose‐5‐phosphate; S7P: Sedoheptulose‐7‐phosphate; E4P: Erythrose‐4‐phosphate; TCA: Tricarboxylic acid cycle; MIT: Mitochondria; ER: Endoplasmic reticulum; PER: Peroxisome; LB: Lipid body; HXK1: Hexokinase 1; ZWF1: Glucose‐6‐phosphate dehydrogenase; PGL: 6‐phosphogluconolactonase; GND1: 6‐phosphogluconate dehydrogenase; TKL1: Transketolase 1; TAL: Transaldolase; PGI: Phosphoglucose isomerase; PFK1: Phosphofructokinase 1; FBP1: Fructose‐1,6‐bisphosphatase 1; GAPDH: Glyceraldehyde 3‐phosphate dehydrogenase; PGK: Phosphoglycerate kinase; PCK1: Phosphoenolpyruvate carboxykinase 1; GPD1: Glycerol‐3‐phosphate dehydrogenase 1; SCT1: Glycerol‐3‐phosphate acyltransferase; SLC1: Lysophospholipid acyltransferase; PAH1: Phosphatidic acid phosphohydrolase 1; DGA1/DGA2: Diacylglycerol acyltransferase 1/2; FAS1/FAS2: Fatty acid synthase 1/2; ACC1: Acetyl‐CoA carboxylase 1; MAE: Malic enzyme; MLS1: Malate synthase 1; CIT1: Citrate synthase 1; MDH1/MDH2: Malate dehydrogenase 1/2; ICL2: Isocitrate lyase 2; IDP2: Isocitrate dehydrogenase 2; ACL1: ATP citrate lyase 1; TbFRD: Fumarate reductase derived from *Trypanosoma brucei*; POX1/POX2/POX4/POX5: Acyl‐CoA oxidases; MFE1: Multifunctional enzyme 1 (peroxisomal); POT1: 3‐ketoacyl‐CoA thiolase; PEX10: Peroxisomal biogenesis factor 10.

To determine how cellular processes were reprogrammed and xylose metabolism was enhanced, GO term enrichment analysis was performed on strain EX413 in comparison with strain Hi‐SA0‐X4. The results showed that significantly upregulated terms were functionally dispersed, while significantly downregulated terms were concentrated in the categories related to central metabolism, including carbohydrate transport, fatty acid oxidation, tricarboxylic acid cycle, and glyoxylate cycle (**Figure** **3**a; Figure S6, Supporting Information). Furthermore, Kyoto Encyclopedia of Genes and Genomes (KEGG) pathway enrichment analysis revealed significant downregulation of pathways associated with carbohydrate metabolism, transport and catabolism, fatty acid metabolism, amino acid metabolism, and metabolism of cofactors and vitamins, potentially regulated by Snf1 and Scp1 (Figure 3b).

As shown in Figure 3c, the expression levels of genes involved in central metabolism, including fatty acid oxidation, glycolysis, and glyoxylate shunt, were significantly downregulated. This result was consistent with our observation that glucose utilization and SA biosynthesis in glucose medium were impaired in the evolved strain EX413. Furthermore, genes such as *XDH* and *TKL1*, which are involved in xylose catabolism, were upregulated in strain EX413. This suggests a possible increase in metabolic flux through the oxidoreductase pathway and the non‐oxidative PPP, facilitating the conversion of xylose into glycolytic intermediates. Overall, we speculate that the reduced flux through downstream metabolic pathways could contribute to the ability of evolved strains to use xylose.

### 2.5 Multi‐Copy Expression of *XR*, *XDH*, and *XK* Genes Restores Xylose Catabolic Capacity

**Figure 4:**
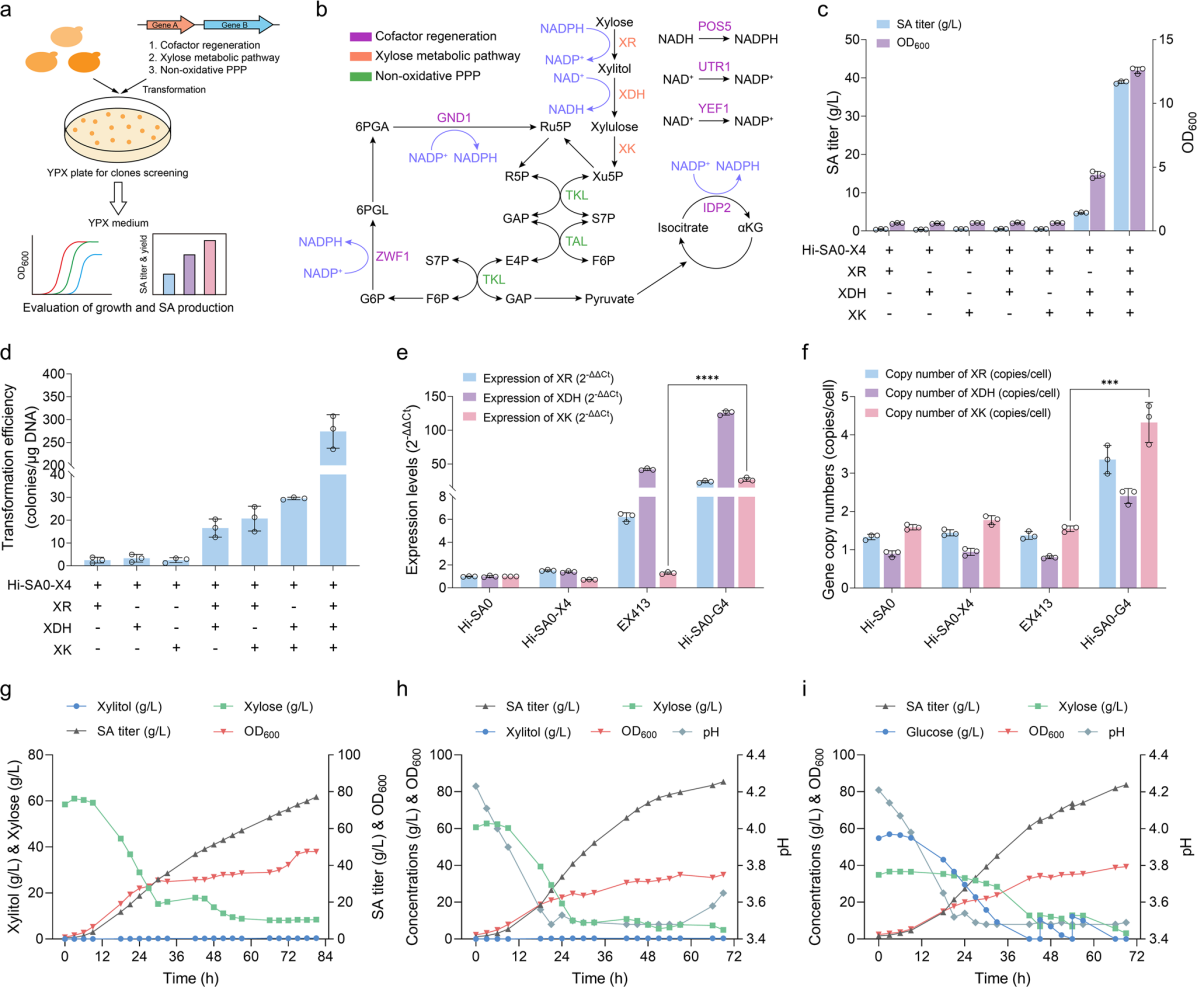
Multiple copy expression of xylose catabolism pathway genes in the strain Hi‐SA0‐X4 for enhanced xylose utilization and SA production. a) Schematic representation of the growth‐coupled random expression library screening approach. b) Schematic illustration of the xylose assimilation module. 6PGA: 6‐Phosphogluconate; Ru5P: Ribulose‐5‐phosphate; R5P: Ribose‐5‐phosphate; Xu5P: Xylulose‐5‐phosphate; 6PGL: 6‐Phosphogluconolactone; G6P: Glucose‐6‐phosphate; S7P: Sedoheptulose‐7‐phosphate; E4P: Erythrose‐4‐phosphate; F6P: Fructose‐6‐phosphate; GAP: Glyceraldehyde‐3‐phosphate; αKG: Alpha‐Ketoglutarate; POS5: NADH kinase; UTR1: NAD kinase; YEF1: ATP‐NADH kinase; GND1: 6‐phosphogluconate dehydrogenase; ZWF1: Glucose‐6‐phosphate dehydrogenase; TKL: Transketolase; TAL: Transaldolase; IDP2: Isocitrate dehydrogenase. c) SA production from xylose by engineered strains with different combinations of xylose metabolic pathway genes. d) Transformation efficiency of different combinations of genes involved in the xylose metabolic pathway. e) Expression levels and f) gene copy numbers of xylose metabolic pathway genes in engineered strains. g) Fed‐batch fermentation profiles of Hi‐SA0‐G4 in YPX medium without pH adjustment. Fed‐batch fermentation profiles of Hi‐SA0‐G4 in h) CM1X medium and i) CM1H medium at pH 3.5. Error bars represent mean ± s.d. (*n* = 3 biologically independent samples). Statistical analysis was performed using a two‐tailed Student's *t*‐test (***p < 0.001, ****p < 0.0001).

While adaptive evolution restored xylose utilization in strain EX413, its reduced glucose metabolism and SA biosynthesis rendered it unsuitable for industrial‐scale production. To determine whether carbon flux bottlenecks or redox imbalance is the primary constraint for xylose utilization, we developed a random expression library screening strategy in *Y. lipolytica*, leveraging its native non‐homologous end joining (NHEJ) repair mechanism for efficient gene integration (**Figure** **4**a). DNA fragments encoding NADPH regeneration (*ZWF1*, *GND1*, *IDP2*, *POS5*, *UTR1*, and *YEF1*), nonoxidative PPP (*TAL1*, *TKL1*, and *TKL2*), and xylose pathway (*XR*, *XDH*, *XK*) genes were individually or combinatorially transformed into strain Hi‐SA0‐X4. Positive clones were selected on xylose agar plates. Unexpectedly, only co‐transformation of *XR*, *XDH*, and *XK* restored robust xylose utilization and SA production (Figure 4c), despite previous overexpression of these genes failing to activate catabolism (Figure 1b,c).

Furthermore, we observed that the transformation efficiency of different gene combinations in the xylose metabolic pathway varied on xylose plates. The highest transformation efficiency (≈274 colonies per µg DNA) was achieved by co‐introducing the complete xylose metabolic pathway genes (i.e., *XR*, *XDH*, and *XK*), while single‐gene transformations resulted in low efficiency (Figure 4d). Notably, introducing the complete xylose metabolic pathway to generate strain Hi‐SA0‐G4 resulted in superior SA production, achieving a maximum titer of 38.88 g L^−1^ within 72 h and an SA yield of 0.79 g g^−1^ xylose, consistent with the observed transformation efficiency trends (Figure 4c; Figure S7, Supporting Information). Importantly, strain Hi‐SA0‐G4 retained effective glucose utilization and could co‐ferment glucose and xylose for efficient SA production (Figure S7, Supporting Information).

To determine whether insufficient flux in the xylose metabolic pathway hindered xylose utilization, we evaluated the changes in expression levels and gene copy numbers of *XR*, *XDH*, and *XK*. In the engineered strain Hi‐SA0‐G4, higher expression levels were correlated with increased gene copy numbers. The gene copy numbers for *XR*, *XDH*, and *XK* were up to 3.36, 2.40, and 4.32 copies per cell, respectively, with corresponding increases in gene expression levels by 23‐fold, 125‐fold, and 26‐fold (Figure 4e,f). In contrast, the evolved strain EX413 exhibited minimal changes in gene copy numbers but significant increases in expression levels of *XR* and *XDH*. These results highlight that gene dosage of *XR*, *XDH*, and *XK*, rather than the mere presence of the pathway, is critical for overcoming metabolic bottlenecks and enabling efficient xylose utilization in *Y. lipolytica*.

In fed‐batch fermentation using xylose as the sole carbon source, the evolved strain EX413 accumulated a substantial amount of xylitol (8.03 g L^−1^) and produced only 27.20 g L^−1^ SA, indicating that the downstream metabolism of xylitol may be impeded (Figure S8, Supporting Information). In contrast, the engineered strain Hi‐SA0‐G4 rapidly metabolized xylose, producing 77.17 g L^−1^ SA with minimal xylitol byproduct (Figure 4g). Using a cost‐effective modified CM1 medium instead of the expensive YP medium further improved SA productivity (Figure S9a, Supporting Information). Maintaining the pH at 3.5 increased the SA titer to 85.48 g L^−1^ (Figure 4h). Subsequently, we evaluated the performance of strain Hi‐SA0‐G4 in detoxified lignocellulosic hydrolysate. Initial shake flask experiments using 16% (v/v) hydrolysate in CM1H medium demonstrated robust growth (OD_600_ > 18) and an SA titer of 73.08 g L^−1^ (Figure S10, Supporting Information). In fed‐batch fermentation, strain Hi‐SA0‐G4 efficiently utilized both xylose and glucose to produce SA without exhibiting the post‐glucose effect (Figure S9b, Supporting Information), commonly observed in xylose‐fermenting *S. cerevisiae*.^[^ ^40^ ^]^ When the pH was maintained at 3.5 during the fermentation, the SA titer increased to 83.78 g L^−1^ (Figure 4i). These results show that the xylose‐fermenting *Y. lipolytica* strain Hi‐SA0‐G4 has promise for industrial applications in sustainable SA production from lignocellulosic hydrolysates.

### 2.6 Exploring the Effects of Fatty Acid Metabolism on Xylose Utilization and SA Biosynthesis

**Figure 5:**
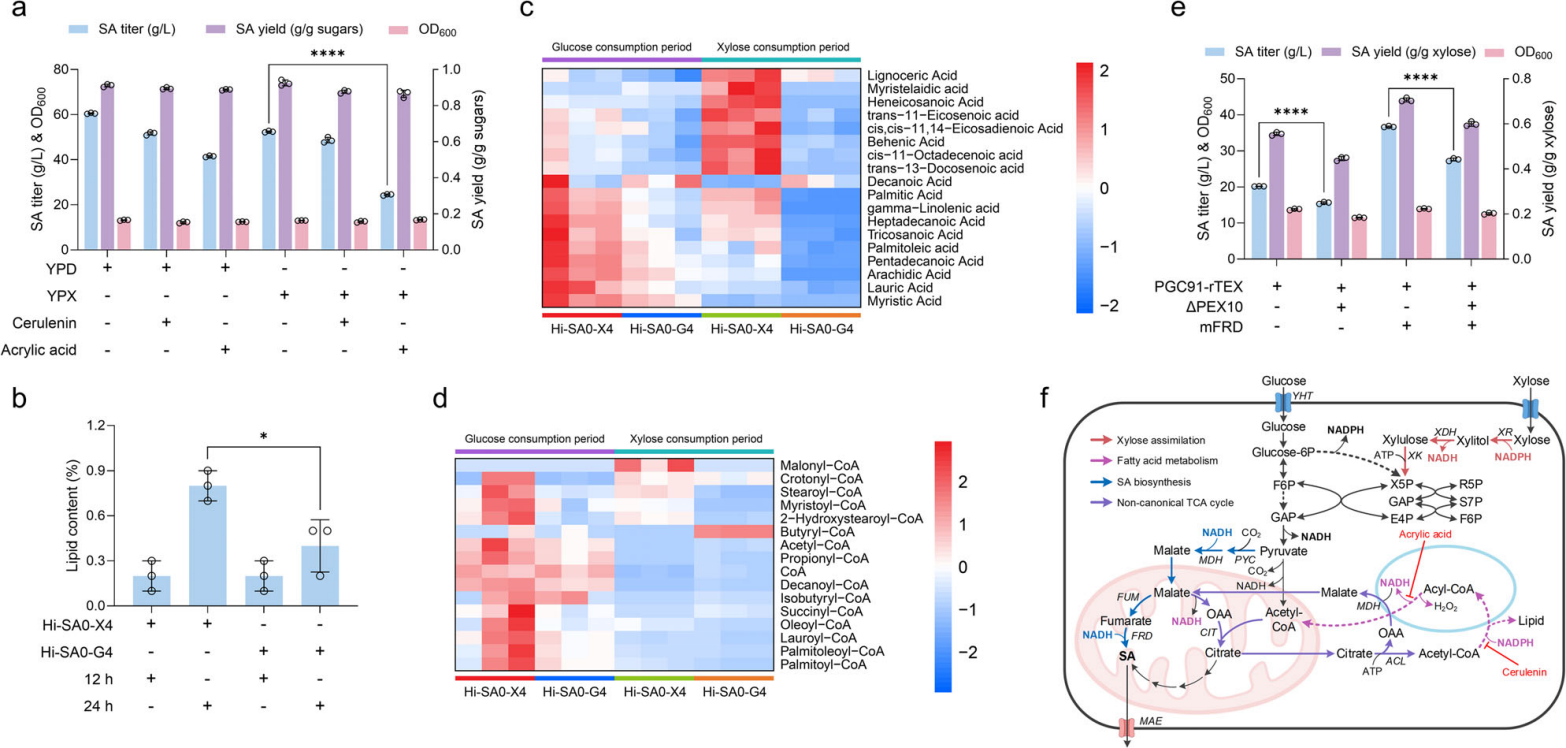
Effects of fatty acid metabolism on xylose assimilation and SA biosynthesis. a) Comparison of SA titer, SA yield, and cell growth (OD_600_) of the engineered strain Hi‐SA0‐G4 in YPD or YPX medium supplemented with cerulenin or acrylic acid. b) Comparison of lipid content of the engineered strain Hi‐SA0‐G4 and the control strain Hi‐SA0‐X4 in YPDX medium. c) Heatmap of the top 18 metabolites in fatty acid metabolism for Hi‐SA0‐X4 and Hi‐SA0‐G4 strains during different metabolic stages. d) Heatmap of the top 16 metabolites in acyl‐CoA metabolism for Hi‐SA0‐X4 and Hi‐SA0‐G4 strains during different metabolic stages. In both figures c) and d), the color blocks at different positions represent the relative expression levels of the corresponding metabolites; red indicates high expression in its respective group, while blue indicates low expression. e) The impact of β‐oxidation disruption and enhancement of the mitochondrial rTCA pathway on SA production in xylose medium. f) Metabolic model of how fatty acid metabolism affects SA biosynthesis and xylose catabolism in SA‐producing *Y. lipolytica*. YHT: Glucose transporters; XR: Xylose reductase; XDH: Xylitol dehydrogenase; XK: Xylulokinase; Xu5P: Xylulose‐5‐phosphate; R5P: Ribose‐5‐phosphate; S7P: Sedoheptulose‐7‐phosphate; F6P: Fructose‐6‐phosphate; GAP: Glyceraldehyde‐3‐phosphate; E4P: Erythrose‐4‐phosphate; F6P: Fructose‐6‐phosphate; Glucose‐6P: Glucose‐6‐phosphate; NADH: Nicotinamide adenine dinucleotide NADPH: Nicotinamide adenine dinucleotide phosphate; ATP: Adenosine triphosphate; FUM: Fumarase; FRD: Fumarate reductase; MDH: Malate dehydrogenase; PYC: Pyruvate carboxylase; OAA: Oxaloacetate; CIT: Citrate synthase; ACL: ATP‐citrate lyase; MAE: C4‐dicarboxylic acid transporter. Error bars represent mean ± s.d. (*n* = 3 biologically independent samples). Statistical analysis was performed using a two‐tailed Student's *t*‐test (*p < 0.05, ****p < 0.0001).

The β‐oxidation pathway was significantly downregulated in the evolved strain EX413, and high expression of *XR*, *XDH*, and *XK* genes restored growth using xylose, suggesting that fatty acid metabolism may be involved in the regulation of xylose catabolism in *Y. lipolytica*. To validate this hypothesis, we tested the growth of different xylose‐utilizing strains Po1fX (wild‐type), PGC91‐rTEX (carrying an enhanced cytosolic rTCA pathway), and Hi‐SA0‐G4 (carrying an enhanced mitochondrial rTCA pathway) in YPX medium supplemented with different concentrations of the fatty acid synthase inhibitor cerulenin or the β‐oxidation inhibitor acrylic acid (Figure S11, Supporting Information). Among them, the strain Hi‐SA0‐G4 was highly sensitive to acrylic acid, with a maximum tolerated concentration of only 0.5 g L^−1^. The addition of acrylic acid at 24 h during shake‐flask fermentation inhibited both glucose and xylose metabolism, with xylose metabolism being more severely affected. In YPX medium, the SA titer decreased significantly by 53.3%; however, neither the SA yield nor the final OD_600_ was impacted by acrylic acid treatment (**Figure** **5**a; Figure S12, Supporting Information).

We analyzed the intracellular lipid content of the strain Hi‐SA0‐G4 cultivated in YPDX medium (Figure S13, Supporting Information). During the glucose consumption period (12 h), the lipid content remained relatively unchanged. However, a significant decrease of 50% in lipid content was observed during the xylose consumption period (24 h) compared to the glucose consumption period (Figure 5b). Metabolomic analysis revealed the downregulation of fatty acid metabolism in Hi‐SA0‐G4 during the xylose consumption period, relative to its own glucose consumption period. Throughout both carbon source consumption periods, Hi‐SA0‐G4 consistently exhibited lower levels of fatty acid metabolites compared to the control strain Hi‐SA0‐X4 (Figure 5c). Furthermore, metabolite levels of acyl‐CoA were similar to the changes observed in fatty acids (Figure 5d). These results indicate that reduced fatty acid synthesis or enhanced β‐oxidation occurs in this SA‐producing strain with multi‐copy expression of xylose metabolic pathway genes. Subsequently, the *PEX10* gene, encoding peroxisomal biogenesis factor 10, was deleted in the strain PGC91‐rTEX, leading to decreased SA production in xylose medium (Figure 5e). Similar impairment of SA biosynthesis was observed in the PEX10‐deficient strain overexpressing mitochondrial fumarate reductase (mFRD) (Figure 5e). These results indicate that the xylose‐dependent growth of the SA‐producing strain Hi‐SA0‐G4 relies on fatty acid metabolism, and this trait is associated with the expression of the mitochondrial rTCA pathway. We therefore propose a model wherein the heterologous rTCA cycle interferes with xylose metabolism in *Y. lipolytica* (Figure 5f). Specifically, the fatty acid synthesis‐oxidation futile cycle may drive SA biosynthesis through the conversion of cytosolic NADPH to mitochondrial NADH, while competitively depleting NADPH to suppress xylose catabolism.

### 2.7 Energy Metabolome Analysis Reveals Xylose Metabolic Mechanisms

**Figure 6:**
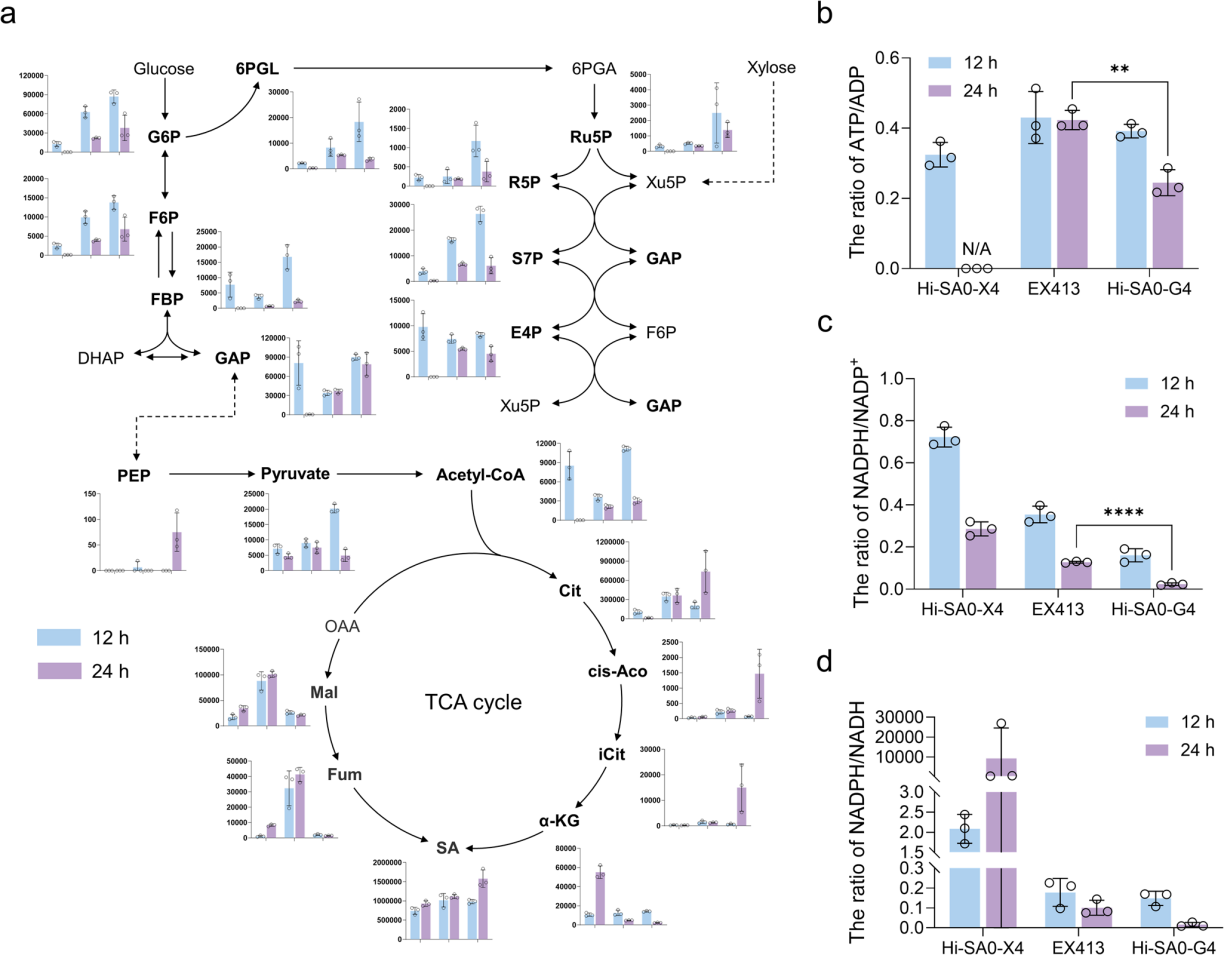
Energy metabolomic analysis for validation of the proposed metabolic model. a) Metabolite profiles in glycolysis, TCA cycle, and PPP for strains Hi‐SA0‐X4, EX413, and Hi‐SA0‐G4. *y‐axis*: Metabolite concentration (nmol L^−1^); *x‐axis*: Strains (left to right: Hi‐SA0‐X4, EX413, Hi‐SA0‐G4). G6P: Glucose‐6‐phosphate; F6P: Fructose‐6‐phosphate; FBP: Fructose‐1,6‐bisphosphate; GAP: Glyceraldehyde‐3‐phosphate; DHAP: Dihydroxyacetone phosphate; PEP: Phosphoenolpyruvate; OAA: Oxaloacetate; Mal: Malate; Cit: Citrate; cis‐Aco: cis‐Aconitate; iCit: Isocitrate; αKG: Alpha‐ketoglutarate; Fum: Fumarate; 6PGL: 6‐Phosphogluconolactone; 6PGA: 6‐Phosphogluconate; Ru5P: Ribulose‐5‐phosphate; R5P: Ribose‐5‐phosphate; Xu5P: Xylulose‐5‐phosphate; S7P: Sedoheptulose‐7‐phosphate; E4P: Erythrose‐4‐phosphate. b) ATP/ADP ratios in strains Hi‐SA0‐X4, EX413, and Hi‐SA0‐G4 during glucose (12 h) or xylose (24 h) consumption periods. c) NADPH/NADP^+^ ratios in strains Hi‐SA0‐X4, EX413, and Hi‐SA0‐G4 during glucose (12 h) or xylose (24 h) consumption periods. d) NADPH/NADH ratios in strains Hi‐SA0‐X4, EX413, and Hi‐SA0‐G4 during glucose (12 h) or xylose (24 h) consumption periods. Error bars represent mean ± s.d. (*n* = 3 biologically independent samples). Statistical analysis was performed using a two‐tailed Student's *t*‐test (**p < 0.01, ****p < 0.0001).

To further validate the proposed metabolic model, we analyzed the levels of key energy metabolites—encompassing glycolysis, the TCA cycle, the PPP, and the ATP/ADP and NADPH/NADP⁺ ratios—in the initial strain Hi‐SA0‐X4, the evolved strain EX413, and the xylose‐utilizing strain Hi‐SA0‐G4. Since Hi‐SA0‐X4 is incapable of xylose utilization, we performed analyses of these strains in YPDX medium supplemented with a low glucose concentration of 5 g L^−1^ (Figure S13, Supporting Information). Changes in these metabolite levels were measured during the glucose or xylose utilization periods. Due to a block in xylose metabolism, negligible levels of upstream glycolysis and PPP metabolites were detected in strain Hi‐SA0‐X4 during xylose utilization. In contrast, strain Hi‐SA0‐G4 exhibited the highest accumulation of these metabolites, followed by strain EX413 (**Figure** **6**a). This metabolite profile suggests that Hi‐SA0‐G4 possesses the highest glycolytic and PPP flux during xylose catabolism. In the rTCA pathway, strain EX413 showed the highest accumulation of the intermediates malic acid and fumaric acid, indicating reduced flux through this pathway, a result consistent with the transcriptome data. Notably, both Hi‐SA0‐X4 and Hi‐SA0‐G4 displayed significantly lower levels of malic acid and fumaric acid accumulation compared to EX413, suggesting stronger rTCA flux in these strains (Figure 6a). Analysis of the ATP/ADP ratio showed that during xylose utilization, Hi‐SA0‐G4 exhibited a 42.1% lower ATP/ADP ratio compared to strain EX413 (Figure 6b). This indicates enhanced oxidative phosphorylation in Hi‐SA0‐G4, and importantly, that the increased mitochondrial rTCA activity did not impose a significant energetic burden. ATP and ADP levels were undetectable in Hi‐SA0‐X4 during xylose utilization, likely due to arrested cell growth. Regarding cofactor metabolism, strain Hi‐SA0‐G4 exhibited a significantly lower NADPH/NADP⁺ ratio than EX413 during xylose utilization, with a reduction of 76.3% (Figure 6c). Concomitantly, the NADPH/NADH ratio also decreased significantly in Hi‐SA0‐G4 (Figure 6d). These shifts collectively indicate there is more efficient NADPH conversion for xylose utilization in Hi‐SA0‐G4. This result further supports our hypothesis that the excess NADPH is diverted, via a β‐oxidation‐mediated futile fatty acid cycle, to generate mitochondrial NADH for SA synthesis.

## 3 Discussion

Efficient xylose utilization is one of the significant challenges in the conversion of lignocellulosic biomass into bio‐based chemicals by industrial yeasts.^[^ ^41^ ^]^ Previously, an SA‐overproducing strain Hi‐SA0 was constructed by introducing the rTCA pathway into the mitochondrial matrix of an SDH‐deficient *Y. lipolytica*.^[^ ^19^ ^]^ To produce SA from lignocellulose‐derived xylose, the endogenous genes of *XR*, *XDH*, and *XK* were overexpressed in the strain Hi‐SA0. However, the resulting strains failed to grow in xylose medium (Figure 1a–c). In contrast, Prabhu et al. successfully restored growth in xylose medium by overexpressing endogenous xylose assimilation genes in the SDH‐deficient *Y. lipolytica* strain.^[^ ^35^ ^]^ More recently, the SDH was successfully disrupted in a xylose‐utilizing *Y. lipolytica* strain BZ, enabling the resulting strain to synthesize SA from xylose.^[^ ^42^ ^]^ Nonetheless, the SA synthetic pathway of these engineered strains relies on the oxidative TCA cycle, which exhibits poor SA production performance. Therefore, we suggest that the redistribution of carbon flux and redox imbalance triggered by the rTCA cycle contribute to the dysfunction of xylose metabolism in the engineered strain with high SA production.

To restore growth using xylose and elucidate the mechanism of metabolic perturbation, adaptive evolution and a multi‐dimensional analysis strategy were performed in this study. After ≈200 generations of passage, five evolved strains with restored growth and xylose consumption were obtained (Figure 1d–g). Genome resequencing and transcriptional analysis identified mutations Snf1^R78W^ and Scp1^delGTC^ in most evolved strains, which caused a global downregulation of central carbon metabolism, particularly affecting glycolysis and β‐oxidation (Figures 2a and 3). In the well‐studied yeasts, Snf1 and Scp1 are known to regulate the transcription of genes related to glucose metabolism, such as those in glycolysis, the TCA cycle, and lipogenesis (Figure 2d). However, their role in perturbing xylose metabolism in *Y. lipolytica* has not been previously reported. In fed‐batch fermentation, the evolved strain EX413 could grow in xylose medium, but its SA titer and yield were only 27.20 g L^−1^ and 0.49 g g^−1^ xylose, respectively. When glucose was used as the carbon source, the strain EX413 exhibited reduced glucose utilization and SA production compared with the starting strain. These results indicate that the overall decreased downstream metabolic flux causes restored growth on xylose and metabolism in SA‐producing strains, but this improvement comes at the cost of reduced SA biosynthesis.

Subsequently, a random expression library screening strategy was used to avoid the impact of prolonged ALE on SA production. Genes involved in the xylose assimilation pathway, the non‐oxidative PPP, and cofactor regeneration were transformed into the strain Hi‐SA0‐X4. Xylose‐utilizing strains were then randomly selected on agar plates with xylose as the sole carbon source (Figure 4a,b). Under selective pressure, multiple copies of genes could be integrated via the NHEJ repair mechanism in *Y. lipolytica*.^[^ ^43^ ^]^ Unexpectedly, only strains co‐transformed with *XR*, *XDH*, and *XK* showed restored xylose utilization and efficient SA production (Figure 4c). This phenomenon was attributed to the high‐level expression of the xylose assimilation pathway, which exhibited up to a 125‐fold increase compared to the control strain Hi‐SA0 (Figure 4e). Therefore, in addition to weakening downstream metabolic pathways, the multi‐copy expression of genes in the xylose assimilation pathway is an effective strategy to relieve disordered xylose metabolism in *Y. lipolytica*.

While *Y. lipolytica* is widely recognized for its lipid‐accumulating prowess (>20% cell dry weight) and engineered fatty acid production,^[^ ^44^ ^]^ the regulatory role of fatty acid metabolism in governing other pathways remains unexplored. We propose a hypothesis that a futile cycle of fatty acid synthesis and β‐oxidation that competitively couples cytosolic NADPH with mitochondrial NADH, thereby driving SA biosynthesis via the rTCA cycle while constraining xylose catabolism (Figure 5f). Specifically, the mitochondrial rTCA pathway consumes two moles of NADH per mole of SA synthesized from pyruvate, whereas glycolysis generates only one mole of NADH. This imbalance is alleviated by mitochondrial NADH derived from β‐oxidation through a non‐canonical TCA cycle.^[^ ^45^ ^]^ In this cycle, mitochondrial citrate is transported to the cytoplasm, where it is catalyzed by ATP‐citrate lyase (ACL) to produce oxaloacetate. This oxaloacetate is then transported back into the mitochondria through the non‐canonical TCA cycle, accompanied by the conversion of cytoplasmic NADH to mitochondrial NADH (Figure 5f). This is demonstrated by the significant decrease in SA titer and yield in xylose‐evolved and PEX10‐deficient strains. Experimental validation using acrylic acid inhibition assays revealed suppressed SA production in strain Hi‐SA0‐G4 (Figure 5a), confirming an enhanced futile cycle that converts NADPH to NADH in SA‐overproducing strains.

Notably, *Y. lipolytica* naturally exhibits high oxidative PPP flux to supply NADPH for lipogenesis.^[^ ^46^ ^]^ In xylose catabolism, the oxidoreductase pathway consumes NADPH via XR while producing NADH via XDH, leading to direct competition with fatty acid metabolism (Figure 5f). Analysis of the energy metabolome revealed that NADPH competition between xylose catabolism and fatty acid synthesis (Figure 6). For strains with mitochondrial‐localized rTCA cycles, we propose the multi‐copy expression of xylose catabolism genes (e.g., *XR*, *XDH*) to competitively consume NADPH and restore xylose utilization. This strategy addresses cofactor competition and aligns metabolic fluxes to optimize SA biosynthesis.

In summary, systematic engineering of *Y. lipolytica* enabled efficient SA production from xylose. Adaptive evolution and multi‐copy expression of xylose assimilation genes restored xylose utilization and cell growth. In fed‐batch fermentation at low pH, the engineered strain Hi‐SA0‐G4 achieved a high SA titer and yield from corn stover hydrolysate, highlighting its industrial potential for SA production from lignocellulosic biomass (Table S6, Supporting Information).

## 4 Experimental Section

### Strains and Culture Media

*Y. lipolytica* strain Po1f was used for mutation validation and growth assays. Individual colonies were initially cultured in 2 mL of YPD medium with overnight incubation at 30 °C and 220 rpm. Cells were then centrifuged at 6000 rpm for 3 min and resuspended in either YNBD or YNBX medium. These resuspended cells were inoculated into 50 mL of fresh medium in 300 mL shake flasks with an initial OD_600_ of 0.1. Cultivation was carried out at 30 °C and 220 rpm. Samples were collected at 12 h intervals for OD_600_ measurement and sugar consumption analysis. *Y. lipolytica* strain Hi‐SA0, which was derived from the previous study, was used for evolutionary engineering and strain construction.^[^ ^19^ ^]^ All the yeast strains are listed in Table S7 (Supporting Information). Detailed compositions for the culture media used in this study are listed in Table S8 (Supporting Information).

### Plasmid Construction

*Escherichia coli* DH5α was used as the host strain for plasmid construction. For gene overexpression, target gene fragments were amplified by PCR from the genomic DNA of *Y. lipolytica* strain Po1f and assembled into the pUC19 vector using the MultiF Seamless Assembly Mix (Cat# RK21020, ABclonal). For gene deletion, 20 bp guide RNA (gRNA) sequences targeting specific gene loci were designed using the CHOPCHOP web tool (https://chopchop.cbu.uib.no/). Corresponding gRNA plasmids containing the designed sequences were then constructed as previously described.^[^ ^47^ ^]^ All plasmids and primer sequences used in this study are listed in Tables S9 and S10 (Supporting Information), respectively.

### Yeast Strain Construction

For all yeast strain engineering, *Y. lipolytica* strains Po1f, PGC91‐rTE1‐1, and Hi‐SA0 were used as background strains. Strains PGC91‐rTE1‐1 and Hi‐SA0 were previously engineered by integrating the rTCA cycle into an SDH‐deficient *Y. lipolytica*.^[^ ^19^ ^]^ Gene overexpression cassettes were integrated through NHEJ. DNA components (promoters, genes, and terminators) for these cassettes were amplified by PCR using Phanta Max Super‐Fidelity DNA Polymerase (Cat#P505, Vazyme). The pCASyl system, a CRISPR‐Cas9 genome editing tool developed for *Y. lipolytica*, was employed for gene deletion.^[^ ^47^ ^]^ This system uses a single plasmid (pCAS1yl or pCAS2yl) to deliver both the Cas9 nuclease and gRNA expression cassettes to target genes, with or without donor DNA. Linearized DNA fragments or plasmids were transformed into *Y. lipolytica* using the Frozen‐EZ Yeast Transformation II kit (Cat#T2001, Zymo Research). Transformed yeast cells were selected on appropriate solid media.

### Adaptive Laboratory Evolution

ALE was employed to enhance xylose utilization in strain Hi‐SA0‐X4. This process was conducted with three independent replicates using 300 mL shake flasks containing 50 mL of YPDX medium. The evolution process comprised two phases, with glucose and xylose concentration gradients established as shown in Table S11 (Supporting Information). In phase I, strain Hi‐SA0‐X4 was initially cultured in YPDX medium (8 g L^−1^ xylose, 32 g L^−1^ glucose) and serially transferred to fresh medium with increasing xylose and decreasing glucose concentrations. In phase II, to promote the utilization of xylose as the sole carbon source, cultures were transferred to YPX medium that only contained 40 g L^−1^ xylose. At the end of ALE, the evolved cultures were plated on YPX agar plates, and 24 colonies from each population were isolated. These colonies were subsequently evaluated for their growth and SA production in YPX medium. The five strains (EX103, EX106, EX205, EX209, and EX413) exhibiting the highest SA production from xylose were further selected for genome resequencing.

### Genome Resequencing

Cultures of the unevolved strain Hi‐SA0‐X4 and five evolved strains were harvested at the exponential growth phase (OD_600_ was ≈8.0) in YPD medium. Genomic DNA was extracted using the TIANamp Yeast DNA Kit (Cat#DP307, TIANGEN), and its quality was validated with an Agilent 2100 Bioanalyzer. For each sample, 200 µg of genomic DNA was randomly fragmented by Covaris to an average size of 300–350 bp. Sequencing was performed on the Illumina HiseqXten/Novaseq/MGI2000 System with 2 × 150 bp paired‐end reads, yielding between 32 and 38 million read pairs per strain. Clean data were mapped to the reference Po1f genome (https://www.ncbi.nlm.nih.gov/nuccore/ML755977.1/) using the Sentieon Pipeline (V202112.02), which also handled duplication removal and SNV/InDel calling. SNV/InDel annotation was performed using Annovar (V21 Apr 2018).^[^ ^48^ ^]^ The genome resequencing was performed by Azenta Life Sciences (Suzhou, China).

### Transcriptome Profiling

Biological triplicates of the parental strain Po1fX and the mutant strains Po1fX‐Snf1^R78W^, Po1fX‐Scp1^delGTC^, and Po1fX‐Rho1^G156S^ were harvested at the exponential growth phase (OD_600_ was ≈8.0) in YPX medium, while biological triplicates of the unevolved strain Hi‐SA0‐X4 and the evolved strain EX413 were harvested at the same growth phase in YPD medium. Total RNA was extracted using TRIzol reagent (Invitrogen), and its purity and concentration were assessed with a NanoDrop 2000 spectrophotometer (Thermo Scientific). RNA quality was validated using an Agilent 2100 Bioanalyzer. RNA libraries were constructed using the VAHTS Universal V6 RNA‐seq Library Prep Kit according to the manufacturer's instructions and sequenced on the Illumina Novaseq 6000 platform by OE Biotech, Inc. (Shanghai, China). All sequencing was performed with 2 × 150 bp paired‐end reads, yielding between 39 and 48 million read pairs per strain. Clean reads were mapped to the reference genome using the HISAT2 alignment software.^[^ ^49^ ^]^ FPKM values and read counts were calculated using the HTSeq‐count tool (v 0.11.2).^[^ ^50^ ^]^ DEGs between samples were analyzed using the DESeq2 (v 1.22.2) method.^[^ ^51^ ^]^ Based on the hypergeometric distribution, GO and KEGG pathway enrichment analyses of DEGs were conducted to identify significantly enriched terms.^[^ ^52^, ^53^ ^]^ The significant enrichment terms were then visualized in a column diagram using R (v 3.2.0).

### SA Production in Shake Flasks

SA production by strain EX413 was conducted in shake flasks containing YP medium with 60 g L^−1^ glucose, 60 g L^−1^ xylose, or a mixture of 30 g L^−1^ glucose and 10 g L^−1^ xylose. Similarly, SA production by strain Hi‐SA0‐G4 was performed in YP medium or CM1 medium with 80 g L^−1^ glucose, 80 g L^−1^ xylose, a mixture of 45 g L^−1^ glucose and 15 g L^−1^ xylose, or 16% (v/v) lignocellulosic hydrolysate. Lignocellulosic hydrolysate was prepared from corn stover by Angel Yeast Co., Ltd, which contains ≈344 g L^−1^ glucose and 223.4 g L^−1^ xylose. The strains were precultured in YPD medium for 24 h and then transferred to 300 mL shake flasks containing 50 mL medium with an initial OD_600_ of 0.5. Fermentation was carried out at 30 °C and 220 rpm for 72–108 h, with samples collected at 12 h intervals for quantification of biomass (OD_600_), sugar consumption rate, and SA titers.

### Fed‐Batch Fermentation for SA Production

Fed‐batch fermentation was conducted in a 5‐L bioreactor (BXBIO) with a working volume of 4 L. The initial batch fermentation medium consisted of YP or CM1 medium containing either 60 g L^−1^ xylose or 16% (v/v) lignocellulosic hydrolysate. Engineered strains, precultured to an OD_600_ of 12–15, were inoculated into the fermentation medium to achieve an initial OD_600_ of ≈2.0. The fermentation conditions were set at 30 °C, with a stirring speed of 400 rpm and an aeration rate of 1.0 vvm. Once the carbon source in the batch medium was nearly depleted, a feeding strategy was initiated by pumping specific substrates into the bioreactor. For fed‐batch fermentation relying on xylose, a solution of 500 g L^−1^ xylose was continuously fed to maintain a residual xylose concentration of 10 g L^−1^. The feeding rate was dynamically adjusted based on substrate consumption. For fed‐batch fermentation using lignocellulosic hydrolysate, once glucose and xylose were nearly depleted, the hydrolysate was supplemented to maintain the carbon source supply. The pH was controlled at 3.5 by the addition of NH_4_OH as needed during fermentation. Samples of the fermentation broth were periodically collected to measure the concentrations of glucose, xylose, xylitol, and SA.

### Quantification of Metabolites

The concentrations of extracellular glucose, xylose, xylitol, and SA were determined using high‐performance liquid chromatography (HPLC). For this process, a 1 mL sample was filtered through a 0.22 µm filter and analyzed on a Shimadzu HPLC system equipped with an Aminex HPX‐87H column (Bio‐Rad) and a Shimadzu refractive index detector. The column temperature was maintained at 35 °C, and the elution was performed for 20 min using 5 mM H_2_SO_4_ as the mobile phase, with a flow rate of 0.6 mL min^−1^.

### Cofactor Measurement

Strains Hi‐SA0‐X4 and EX413 were cultured in YPDX medium containing 5 g L^−1^ glucose and 15 g L^−1^ xylose at 30 °C and 220 rpm. Yeast cells were harvested at the mid‐log phase (≈24 h), and then washed twice with PBS buffer. Intracellular levels of NADH/NAD^+^ and NADPH/NADP^+^ were quantified using the NADH/NAD^+^ assay kit with WST‐8 (Cat#S0175, Beyotime) and the NADPH/NADP^+^ assay kit with WST‐8 (Cat#S0179, Beyotime) according to the manufacturer's instructions.

### qPCR Analysis

qPCR was employed to detect the transcriptional levels and gene copy numbers of *XR*, *XDH*, and *XK* in engineered strains. For transcriptional level detection, total RNA was extracted using Trizol reagent and reverse‐transcribed into cDNA using the SynScript III RT SuperMix for qPCR (Cat#TSK314S, Tsingke). For gene copy number detection, genomic DNA from the samples was extracted using the Genomic DNA Extraction Kit (Cat#TSP102, Tsingke), followed by a fivefold dilution to serve as a template for qPCR. The qPCR reactions were carried out on a StepOnePlus RT PCR System (Applied Biosystems) using ArtiCan^CEO^ SYBR qPCR Mix (Cat#TSE401, Tsingke). Each sample was analyzed in triplicate. Actin (YALI0D08272g) was used as the reference gene, and relative quantification analysis was conducted using the 2^−ΔΔCT^ method.^[^ ^54^ ^]^ For absolute quantification, a standard curve was constructed using serially diluted known standard plasmids (Table S9, Supporting Information) as templates in PCR reactions, plotting their logarithmic copy numbers against the obtained CT values; the copy number of an unknown sample was subsequently calculated from its CT value in relation to this curve.

### Transformation Efficiency Measurement

Transformation of *Y. lipolytica* was performed using a yeast transformation kit, and positive colonies were selected on the corresponding selective agar plates. To calculate transformation efficiency, fresh *Y. lipolytica* cells were transformed with 500 ng of DNA fragments. After 2–3 days of cultivation, the resulting colonies were counted. Transformation efficiency was determined by dividing the number of colonies by the amount of DNA used.

### Fatty Acid Metabolism Inhibition Experiment

Growth monitoring was conducted in either YPX or YPD medium, supplemented with cerulenin or acrylic acid to inhibit fatty acid synthase and β‐oxidation, respectively. To determine the optimal concentrations of these fatty acid metabolism inhibitors, single colonies of strains Po1fX, PGC91‐rTEX, and Hi‐SA0‐G4 were cultured in 2 mL of YPD medium and incubated at 220 rpm overnight. A 20 µL aliquot of the preculture was then transferred to 1 mL of fresh YPX medium in 24‐well plates, each containing varying concentrations of the inhibitors. Cell growth was quantified using a multi‐detection microplate reader (Synergy HT, Biotek, USA). For shake flask cultivation, strains were precultured in YPD medium overnight and subsequently inoculated into 300 mL shake flasks containing 50 mL of YPD or YPX medium, with an initial OD_600_ of 0.5. To inhibit fatty acid synthesis, 2 mg L^−1^ of cerulenin was added at the start of cultivation, while 0.5 g L^−1^ of acrylic acid was added to inhibit β‐oxidation after 24 h of cultivation. The cultures were incubated for 72 h at 30 °C and 220 rpm, with samples taken every 12 h to assess biomass (OD_600_), sugar consumption, and SA production.

### Measurement of Fatty Acid Composition and Lipid Content

For fatty acid composition measurement, targeted fatty acid metabolomic profiling was performed by Shanghai Biotree Biotech Co., Ltd. In brief, samples were lysed by adding 150 µL water and vortexing for 30 s, followed by three freeze‐thaw cycles. Following lysis, the samples were sonicated for 10 min in an ice‐water bath. For lipid extraction, 400 µL extraction solvent (isopropanol: n‐hexane, 2:3 v/v, containing 0.22 mg L^−1^ stearic‐d35 acid as the internal standard) was added. The mixture was vortexed for 30 s and then sonicated for 10 min in an ice‐water bath. After centrifugation at 12000 rpm for 15 min at 4 °C, the supernatant was transferred to a new 2 mL tube and dried under a nitrogen blow. For derivatization, 500 µL of methanol: trimethylsilyl diazomethane solution (1:2, v/v) was added, and the mixture was incubated at room temperature for 30 min. The derivatized sample was then dried under a nitrogen blow. The residue was reconstituted in 160 µL of n‐hexane. After centrifugation at 12000 rpm for 1 min, the supernatant was transferred to an autosampler vial for GC‐MS analysis.

For intracellular lipid content measurement, a 50 mL yeast culture was centrifuged at 4000 rpm for 10 min to pellet the cells. The pellet was resuspended in distilled water, centrifuged again, and washed twice to remove residual medium. Washed cells were transferred to a pre‐weighed tube, dried to constant weight at 60 °C for 24 h, and the dry cell mass (m_cell) was recorded. The dried cells were hydrolyzed with 10 mL of 8 mol L^−1^ HCl at 80 °C for 1 h. After cooling, lipids were extracted by adding 30 mL diethyl ether, vortexing vigorously for 30 s, and centrifuging at 4000 rpm (4 °C) for 5 min. The ether supernatant was transferred to a pre‐weighed flask (m_container). This extraction was repeated twice, pooling all supernatants. Combined extracts were dried under a nitrogen stream, and the total mass (m_total) was recorded. Lipid content was calculated as: Lipid Content (%) = [(m_total – m_container) / m_cell] × 100%.

### Acyl‐CoA Measurement

Targeted acyl‐CoA metabolomic profiling was performed by Shanghai Biotree Biotech Co., Ltd. In brief, samples were homogenized by adding 600 µL pre‐cold methanol (‐20 °C), vortexing, and transferring to a 2 mL tube for homogenization (60 Hz, 60 s). The resulting homogenate was transferred to a new 2 mL tube, mixed with 600 µL pre‐cold chloroform (−20 °C), and shaken (1400 rpm, 1 min). Following the addition of 240 µL ultrapure water, samples were incubated on ice for 10 min. Centrifugation was then performed at 4 °C and 12000 rpm for 2 min. 250 µL upper layer was transferred to a new microcentrifuge tube, dried in a vacuum centrifugal concentrator, and reconstituted in 50 µL methanol/water (3:2, v/v). After a brief centrifugation, 35 µL of the clear supernatant was transferred to an autosampler vial for HPLC analysis.

### Analysis of Energy Metabolome

Strains Hi‐SA0‐X4, EX413, and Hi‐SA0‐G4 were cultured in 300 mL shake flasks containing 50 mL of YPDX medium (5 g L^−1^ glucose and 15 g L^−1^ xylose) at 30 °C with 220 rpm agitation. Cells were harvested at two metabolic phases: 12 h (glucose consumption phase) and 24 h (xylose consumption phase). The harvested cultures were centrifuged to obtain cell pellets. Pellets were washed twice with phosphate‐buffered saline (PBS), followed by immediate quenching in liquid nitrogen to arrest metabolic activity. Analysis of the energy metabolome was performed by Shanghai Biotree Biotech Co., Ltd. In brief, the sample was diluted with 300 µL water and vortex mixed for 30 s. Two steel beads were added, followed by vortex mixing for 30 s and homogenization by grinding at 35 Hz for 4 min. The sample was then sonicated for 5 min in an ice‐water bath. This combined grinding and sonication cycle was repeated twice. After centrifugation, 250 µL of supernatant was mixed with 750 µL extraction solvent, vortexed for 30 s, and incubated at −40 °C for 1 h. Following centrifugation at 12 000 rpm and 4 °C for 15 min, 900 µL of supernatant was transferred to a 2 mL microcentrifuge tube and dried under vacuum. The residue was reconstituted in 180 µL of a methanol/acetonitrile/water mixture (1:1:2, v/v/v), filtered, and analyzed by UHPLC‐MS/MS.

### Statistical Analysis

All data were presented as mean values ± SD from three independent biological replicates (*n* = 3). The significant differences were analyzed by GraphPad Prism 9.0 Project software using a two‐tailed Student's *t*‐test. A *p*‐value < 0.05 was considered statistically significant (*p < 0.05, **p < 0.01, ***p < 0.001, ****p < 0.0001).

## Conflict of Interest

The authors declare no conflict of interest.

## Author Contributions

Y.Z., Z.C., and Q.Q. conceived the study. Y.Z. designed and performed most of the experiments. X.L., J.H., Z.C., and Q.Q. supervised the project. C.S., J.G., and H.T. assisted with experimental performance. Y.Z., Z.C., and Q.Q. wrote and revised the manuscript.

## Supporting information



Supporting Information

Supplementary Table 1

Supplementary Table 4

Supplementary Table 5

## Data Availability

The data that support the findings of this study are available from the corresponding author upon reasonable request.
