# Supplementary material for: Xylose Metabolism Perturbation in Yarrowia lipolytica for Efficient Succinic Acid Bioproduction from Lignocellulosic Biomass
Source: Adv Sci (Weinh). 2025 Aug 19;12(42):e07999. doi: 10.1002/advs.202507999 (PMC12622525; doi:10.1002/advs.202507999)
Supplement: Supplementary file 1 — Supporting Information [file ADVS-12-e07999-s003.docx]

**Supporting Information for:**

**Xylose Metabolism Perturbation in *Yarrowia lipolytica* for Efficient Succinic Acid Bioproduction from Lignocellulosic Biomass**

Yutao Zhong, Changyu Shang, Jinhong Gu, Huilin Tao, Xuemei Lu, Jin Hou, Zhiyong Cui^*^, Qingsheng Qi^*^

State Key Laboratory of Microbial Technology, Shandong University, Qingdao, 266237, P. R. China

* Corresponding authors.

E-mail: qiqingsheng@sdu.edu.cn (Qingsheng Qi)

cuizhiyong@sdu.edu.cn (Zhiyong Cui)


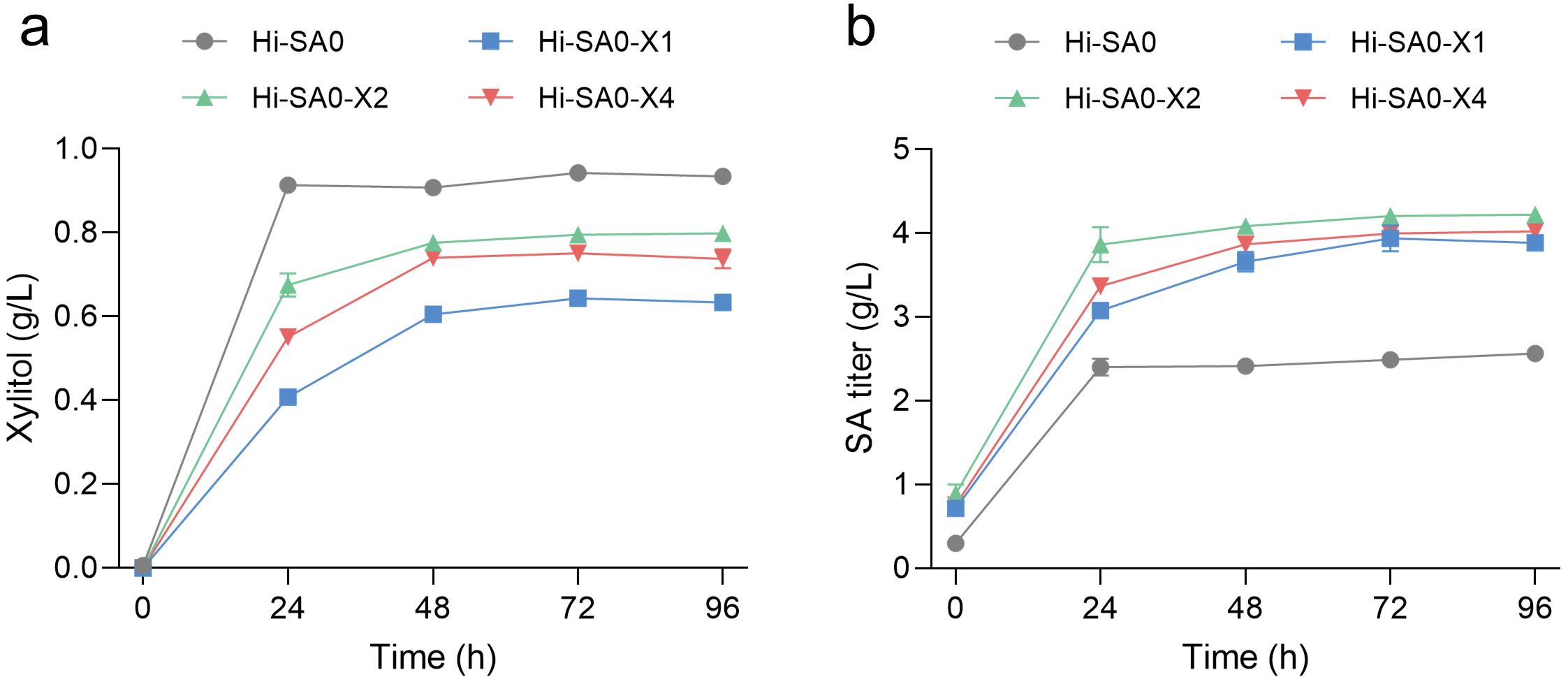


**Figure S1.** Comparison of xylitol formation and SA production in YPX medium by engineered strains Hi-SA0-X1, Hi-SA0-X2, and Hi-SA0-X4. a) Xylitol generation profiles. b) SA titer profiles. Error bars represent mean ± s.d. (n = 3 biologically independent samples).


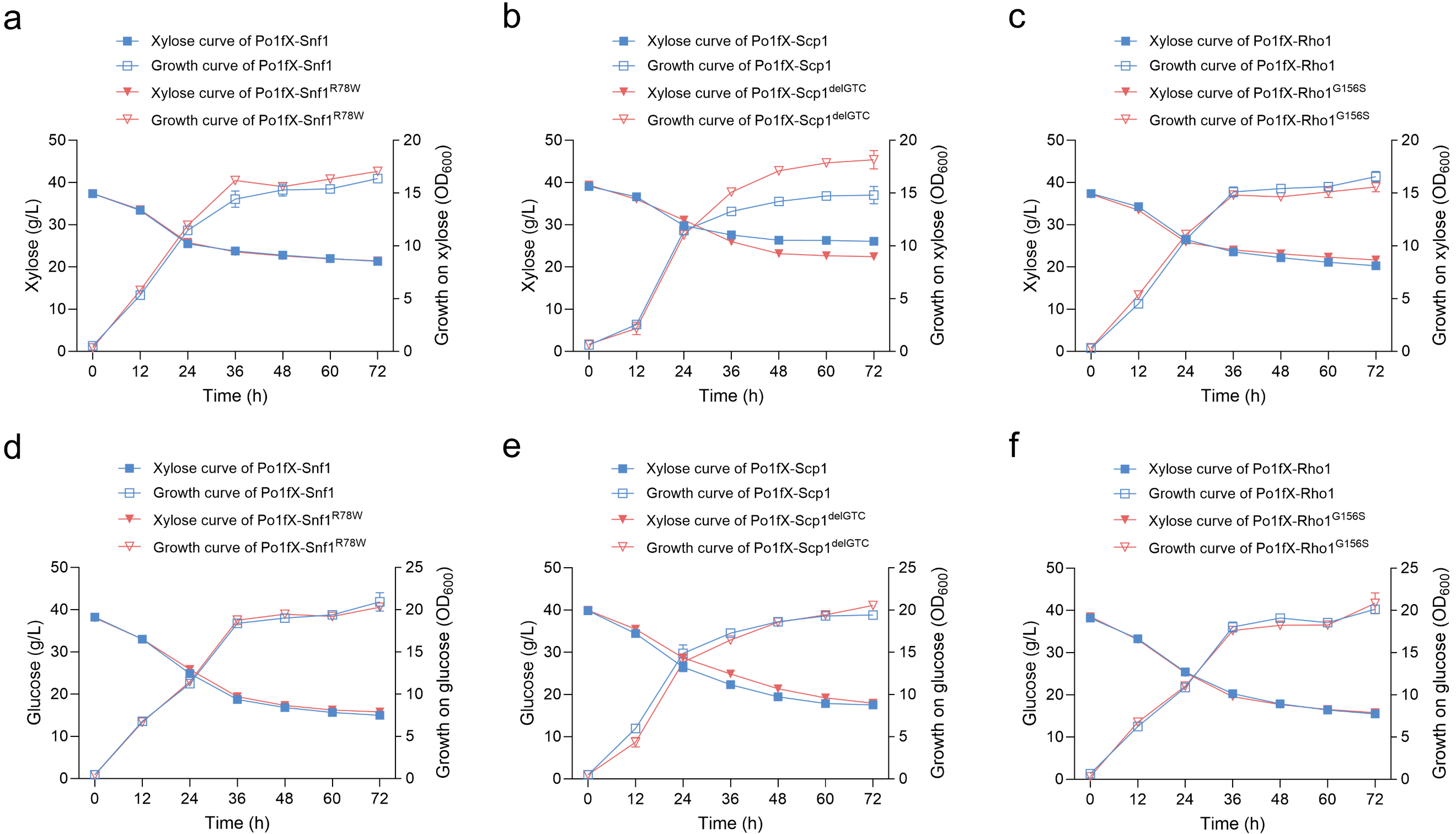


**Figure S2.** Effect of Snf1^R78W^, Scp1^delGTC^, and Rho1^G156S^ on cell growth and sugar consumption of strain Po1fX. Xylose consumption profiles and growth curves (OD_600_) of Snf1 (a), Scp1 (b), and Rho1 (c) mutants in YNBX medium. Glucose consumption profiles and growth curves (OD_600_) of Snf1 (d), Scp1 (e), and Rho1 (f) mutants in YNBD medium. Error bars represent mean ± s.d. (n = 3 biologically independent samples).


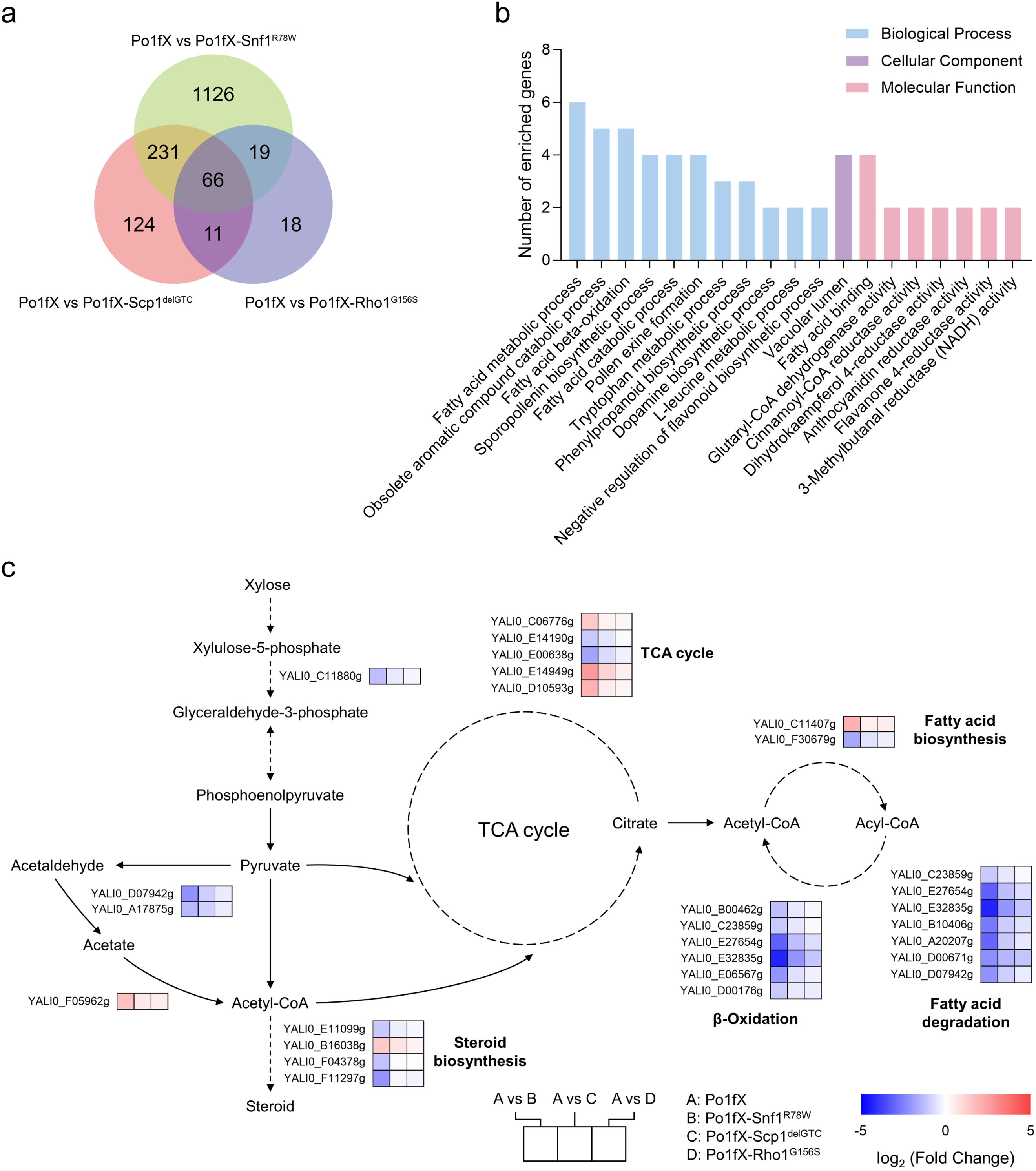


**Figure S3.** Transcriptome analysis of Snf1^R78W^, Scp1^delGTC^, and Rho1^G156S^ mutant strains in xylose medium. a) Venn diagram analysis indicated 66 shared differentially expressed genes (DEGs) between Po1fX and mutant strains, with genes exhibiting a log_2_ fold change ≥ 1 in expression and a q-value ≤ 0.05 defined as DEGs. b) Gene Ontology (GO) analysis was conducted on the 66 enriched DEGs, selecting and visualizing the top 20 most highly enriched GO terms in the categories of biological process (BP), molecular function (MF), and cellular component (CC). c) Transcriptional analysis of key genes in central carbon metabolism.


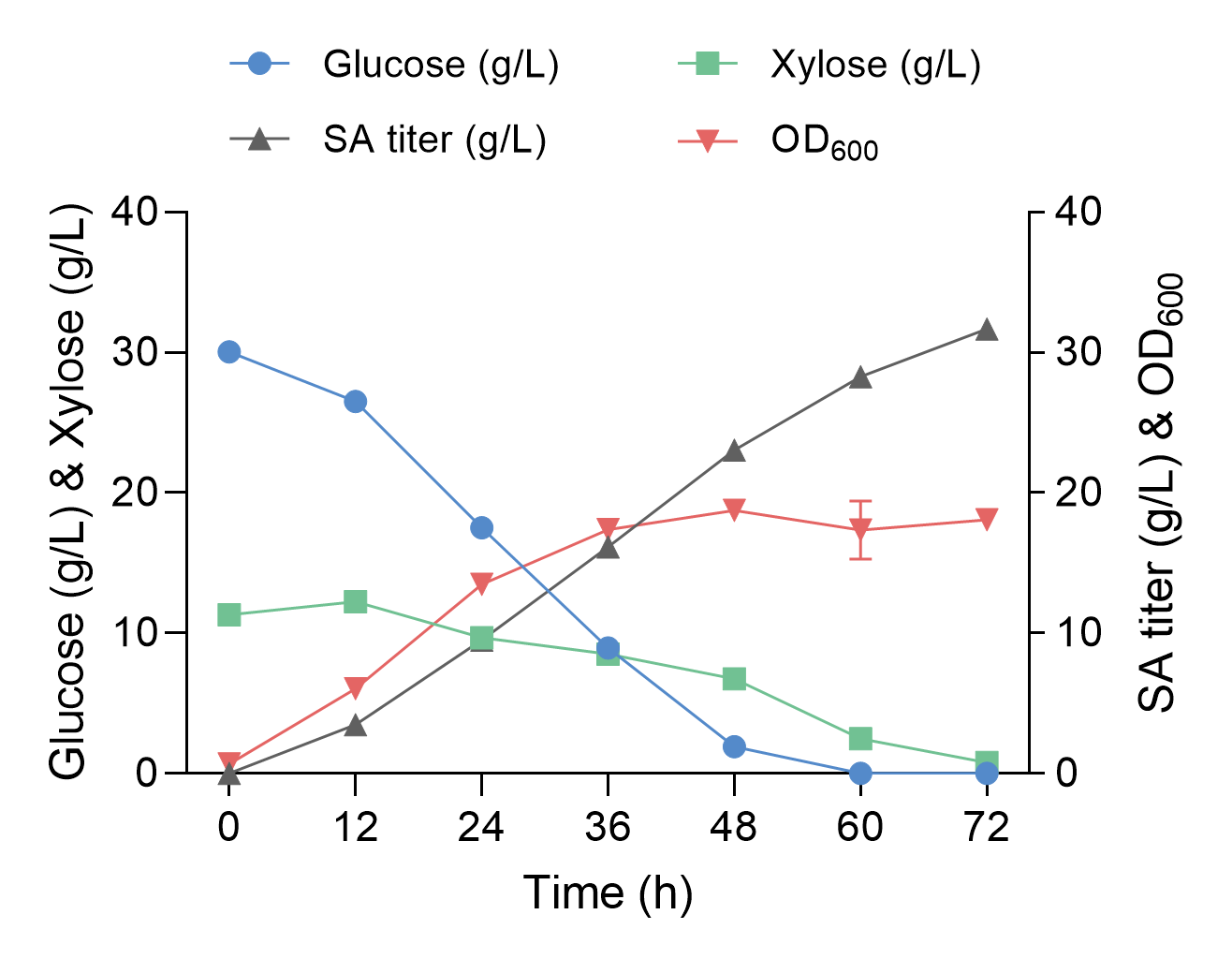


**Figure S4.** Fermentation profiles of the evolved strain EX413 in YPDX medium. Error bars represent mean ± s.d. (n = 3 biologically independent samples).


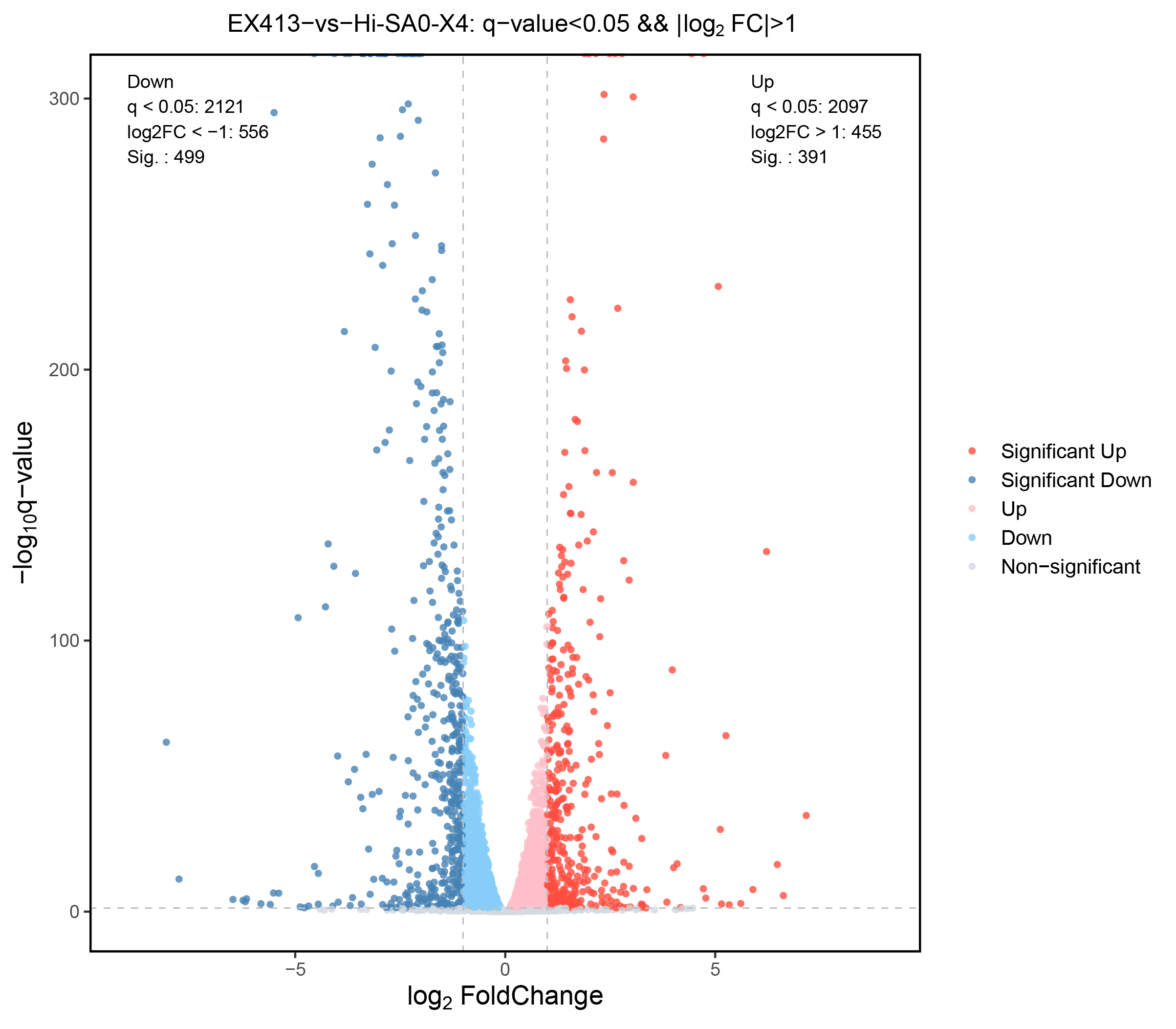


**Figure S5.** Volcano plot of DEGs between the evolved strain EX413 and the unevolved strain Hi-SA0-X4.


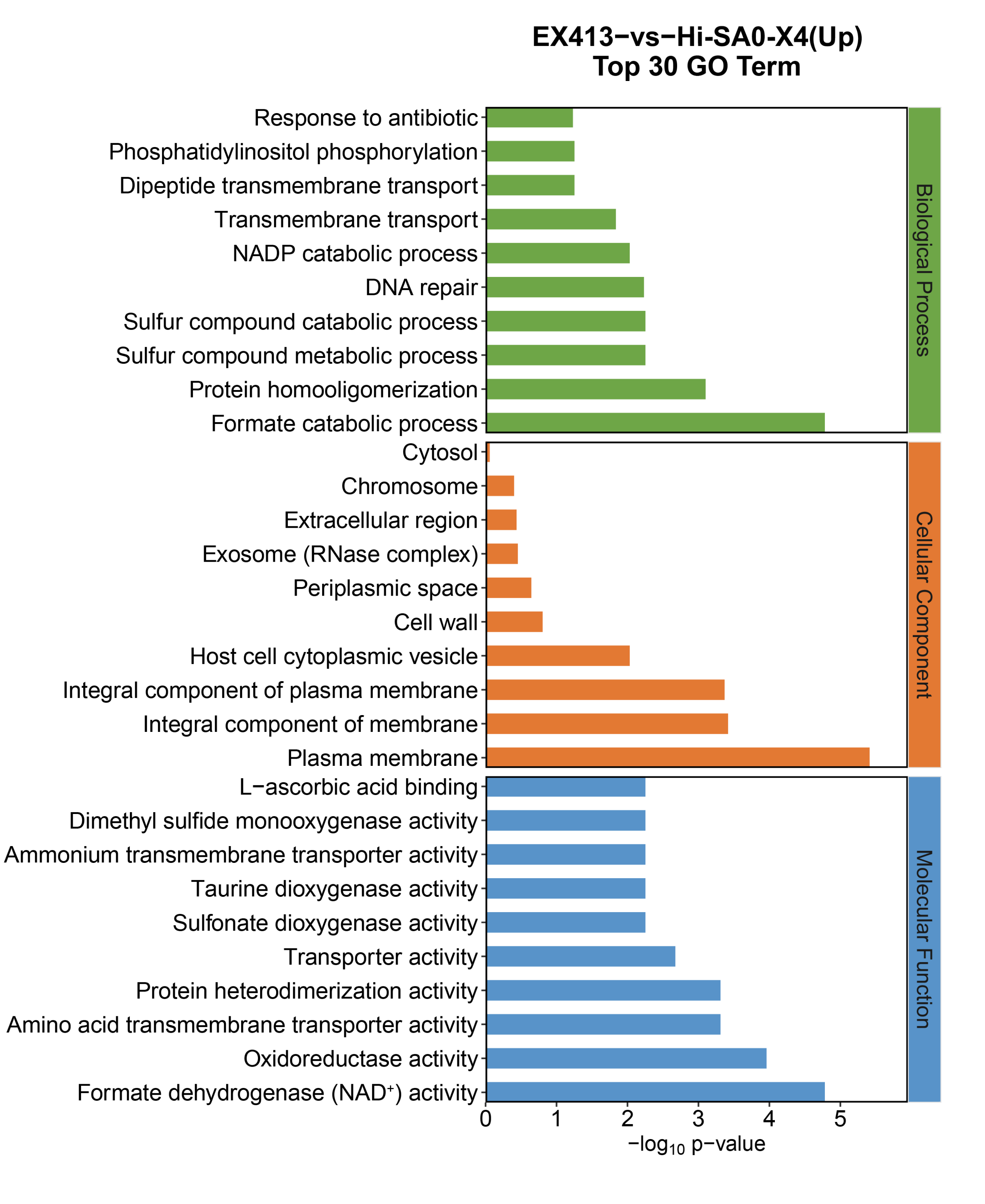


**Figure S6.** Top 30 upregulated GO terms of DEGs between the evolved strain EX413 and the unevolved strain Hi-SA0-X4.


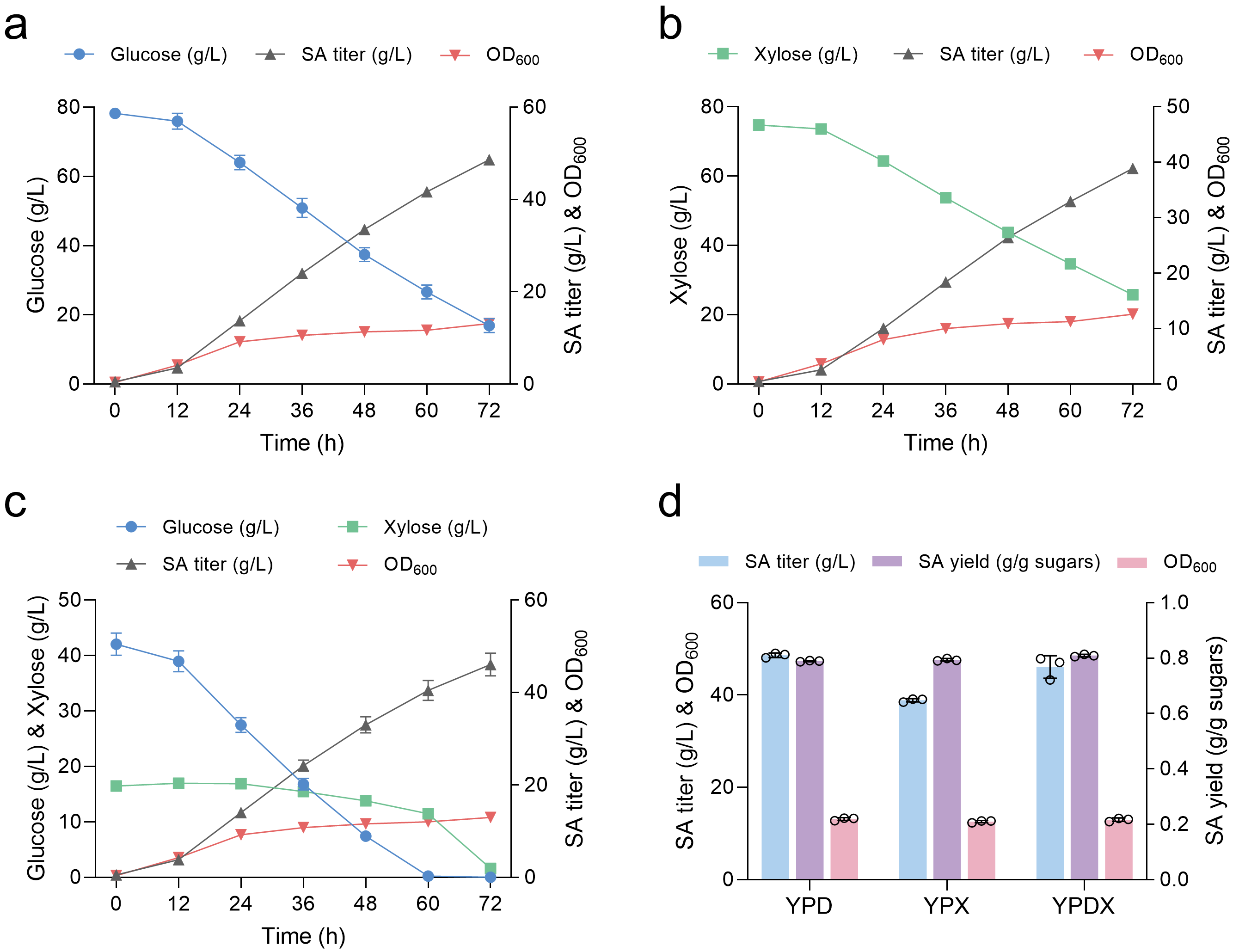


**Figure S7.** Fermentation profiles of the engineered strain Hi-SA0-G4 from different carbon sources. a) Glucose. b) Xylose. c) A mixture of glucose and xylose. d) Comparison of SA titer, SA yield, and OD_600_ within 72 h. Error bars represent mean ± s.d. (n = 3 biologically independent samples).


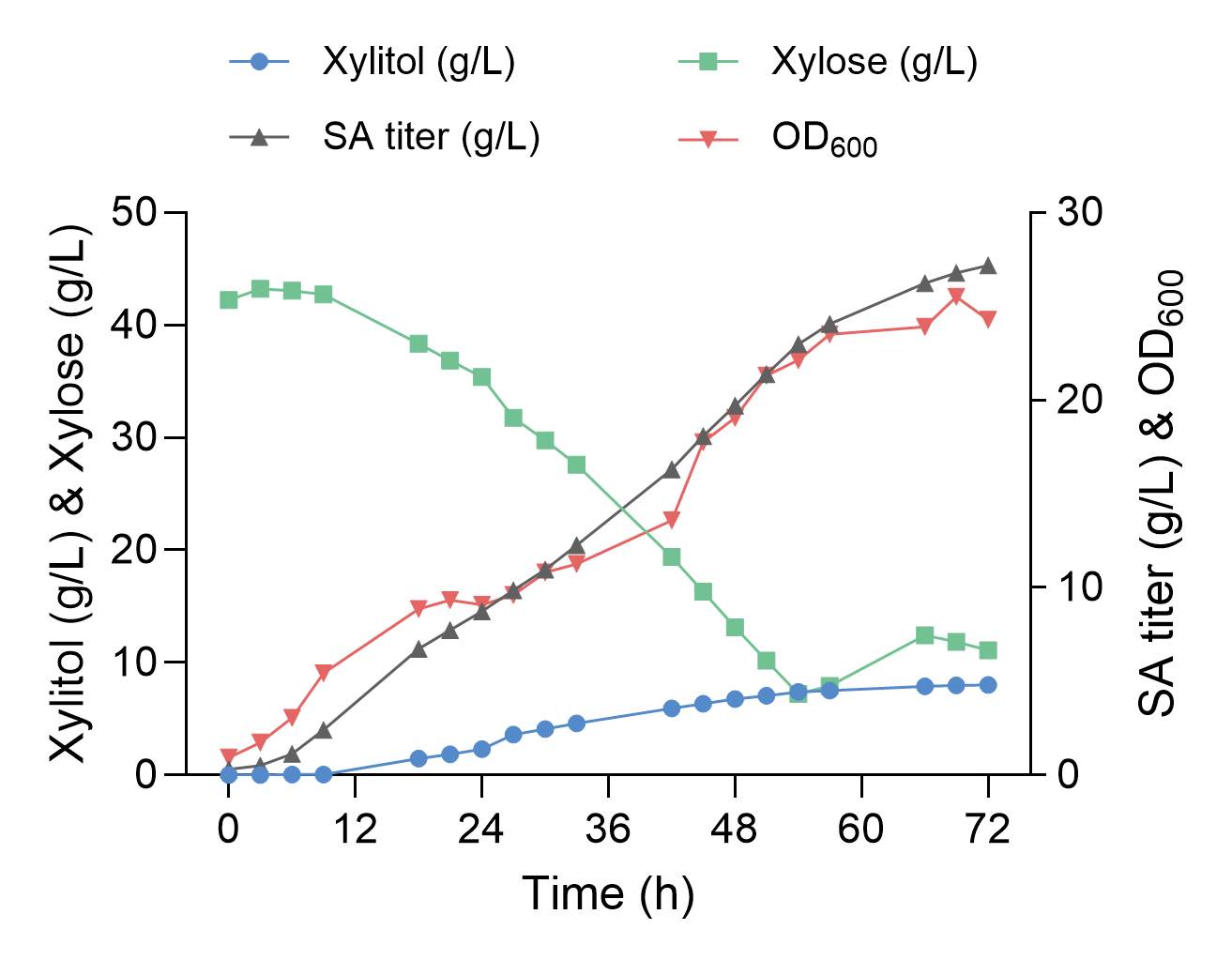


**Figure S8.** Fed-batch fermentation profiles of the evolved strain EX413 in YPX medium without pH adjustment.


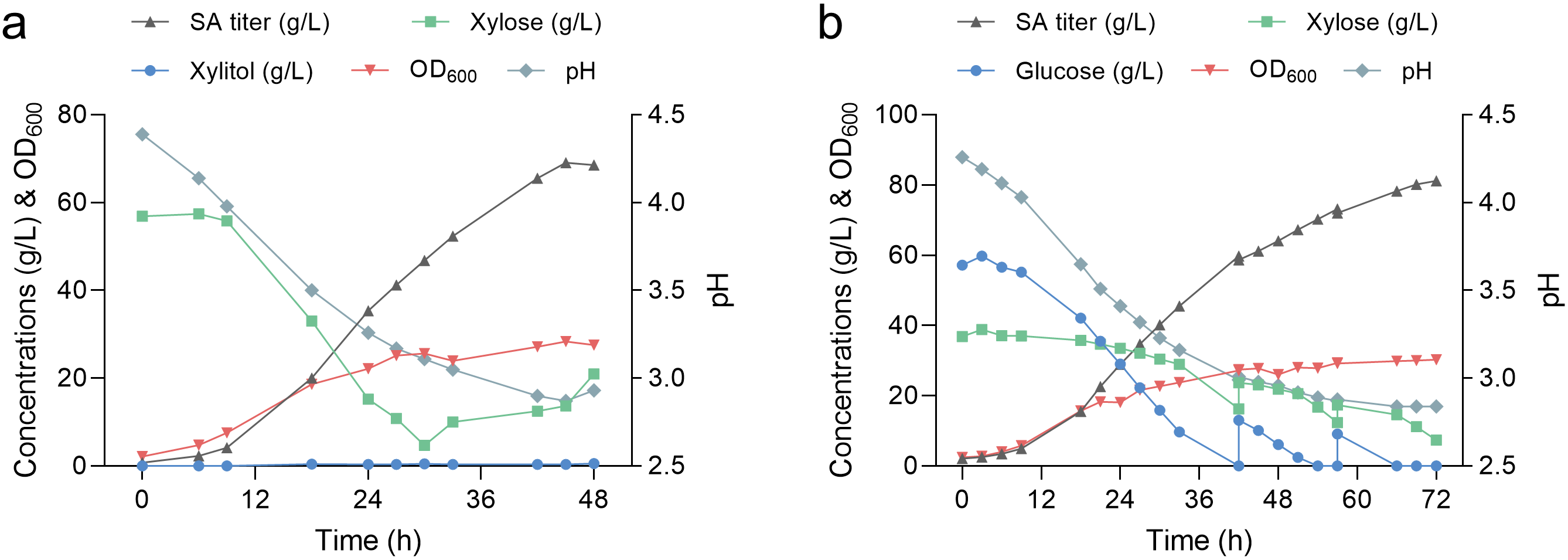


**Figure S9.** Fed-batch fermentation of the engineered strain Hi-SA0-G4 from xylose and corn stover hydrolysate without pH control. a) Fermentation profile in CM1X medium. b) Fermentation profile in CM1H medium supplemented with corn stover hydrolysate.


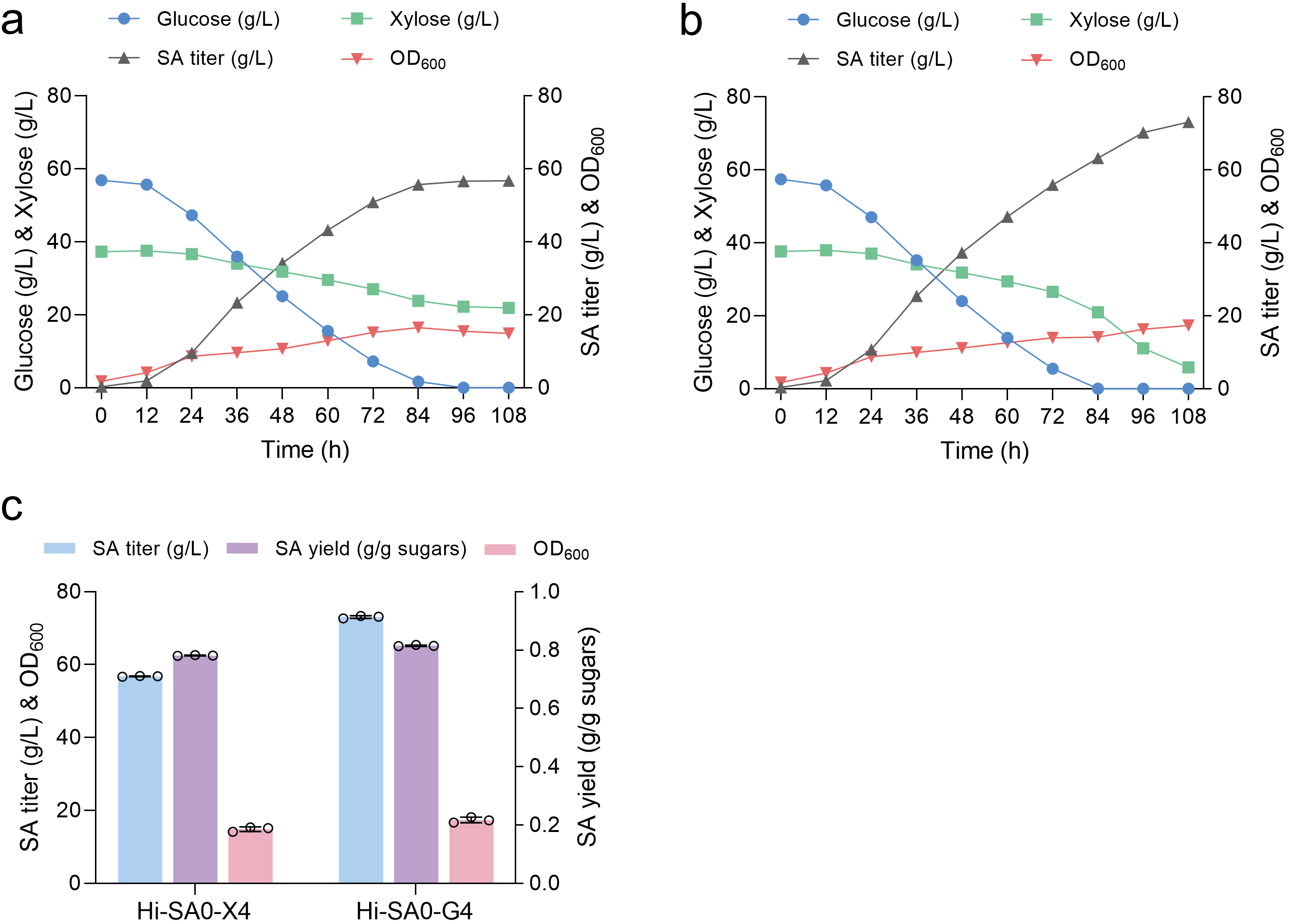


**Figure S10.** SA production from lignocellulosic hydrolysate by engineered *Y. lipolytica* strains. Fermentation profiles of engineered strains Hi-SA0-X4 (a) and Hi-SA0-G4 (b) in shaking flasks using 16% (v/v) corn stover hydrolysate as the sole carbon source. c) Comparison of the SA production performance of engineered strains Hi-SA0-X4 and Hi-SA0-G4 after 108 h of fermentation. Error bars represent mean ± s.d. (n = 3 biologically independent samples).


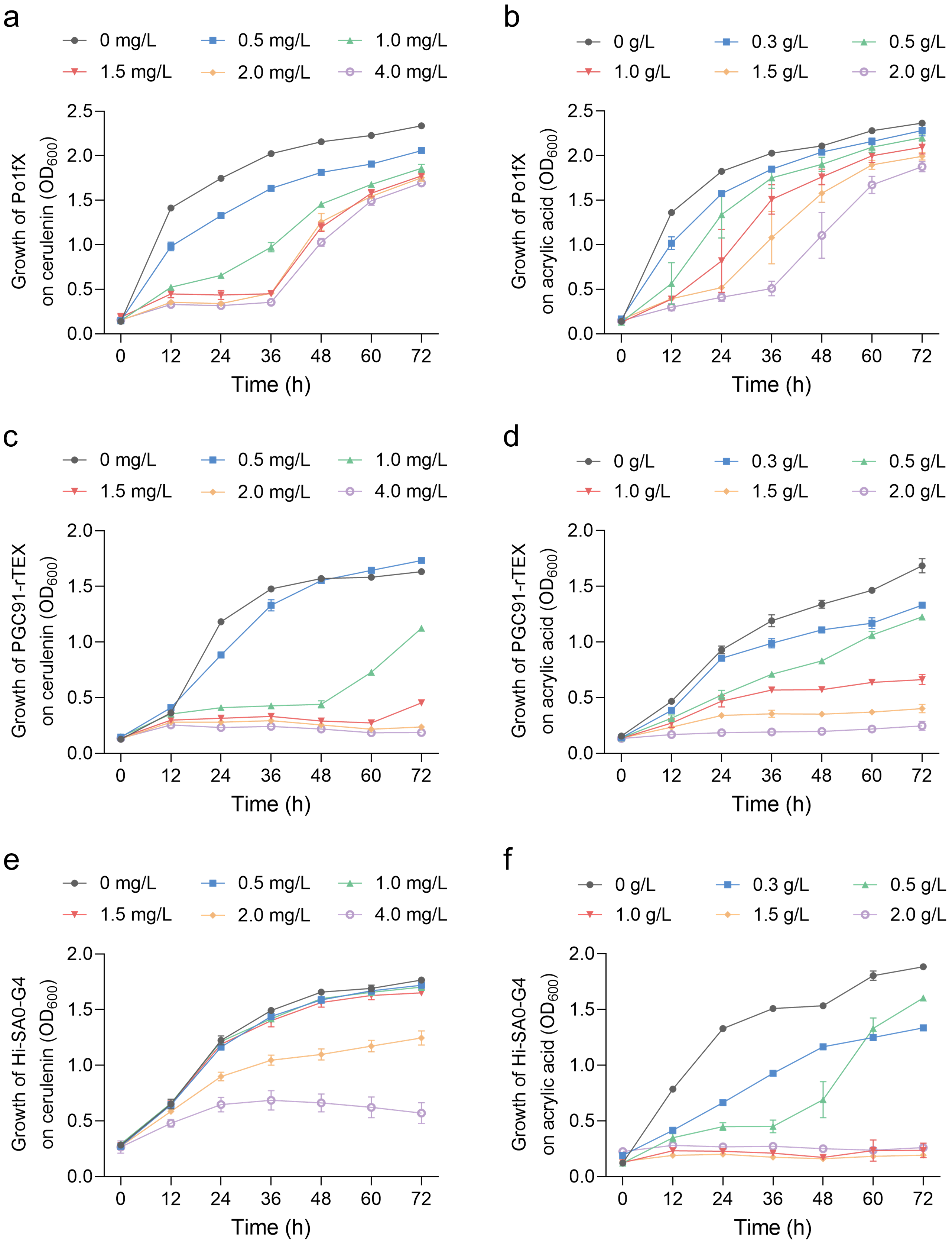


**Figure S11.** Impact of cerulenin and acrylic acid on cell growth in engineered strains. Growth curves of Po1fX under different concentrations of cerulenin (a) and acrylic acid (b) addition. Growth curves of PGC91-rTEX under different concentrations of cerulenin (c) and acrylic acid (d) addition. Growth curves of Hi-SA0-G4 under different concentrations of cerulenin (e) and acrylic acid (f) addition. Po1fX does not harbor functional SA synthetic pathway; PGC91-rTEX harbors a cytosolic reductive TCA pathway for SA biosynthesis; Hi-SA0-G4 harbors a mitochondrial reductive TCA pathway for SA biosynthesis. Error bars represent mean ± s.d. (n = 3 biologically independent samples).


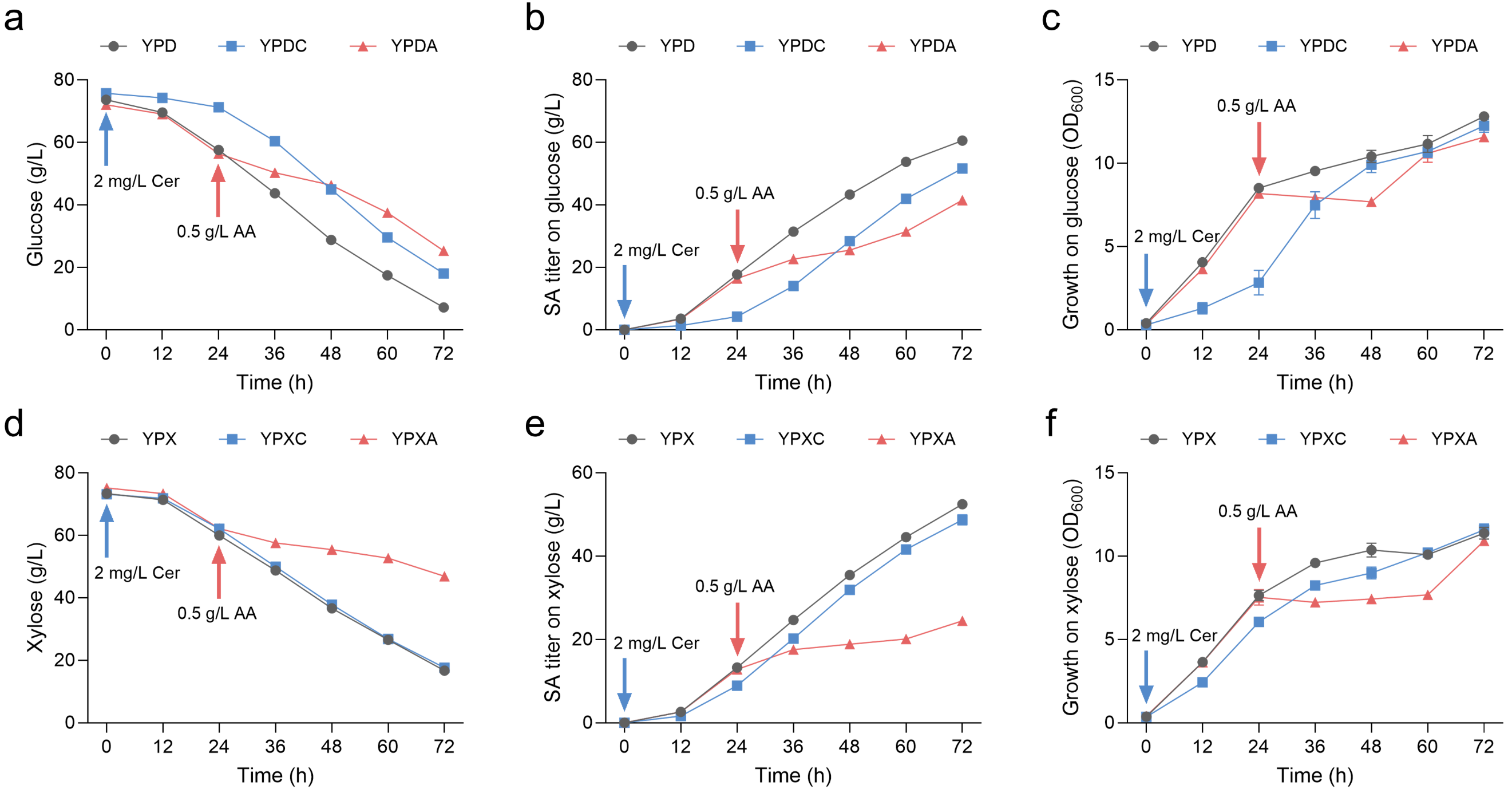


**Figure S12.** Effect of fatty acid metabolism inhibitors on the cell growth and metabolism of SA-producing strain Hi-SA0-G4. Comparison of glucose consumption (a), SA titer (b), and cell growth (c) in YPD medium supplemented with cerulenin or acrylic acid. Comparison of xylose consumption (d), SA titer (e), and cell growth (f) in YPX medium supplemented with cerulenin or acrylic acid. Cer: Cerulenin; AA: Acrylic acid. Error bars represent mean ± s.d. (n = 3 biologically independent samples).


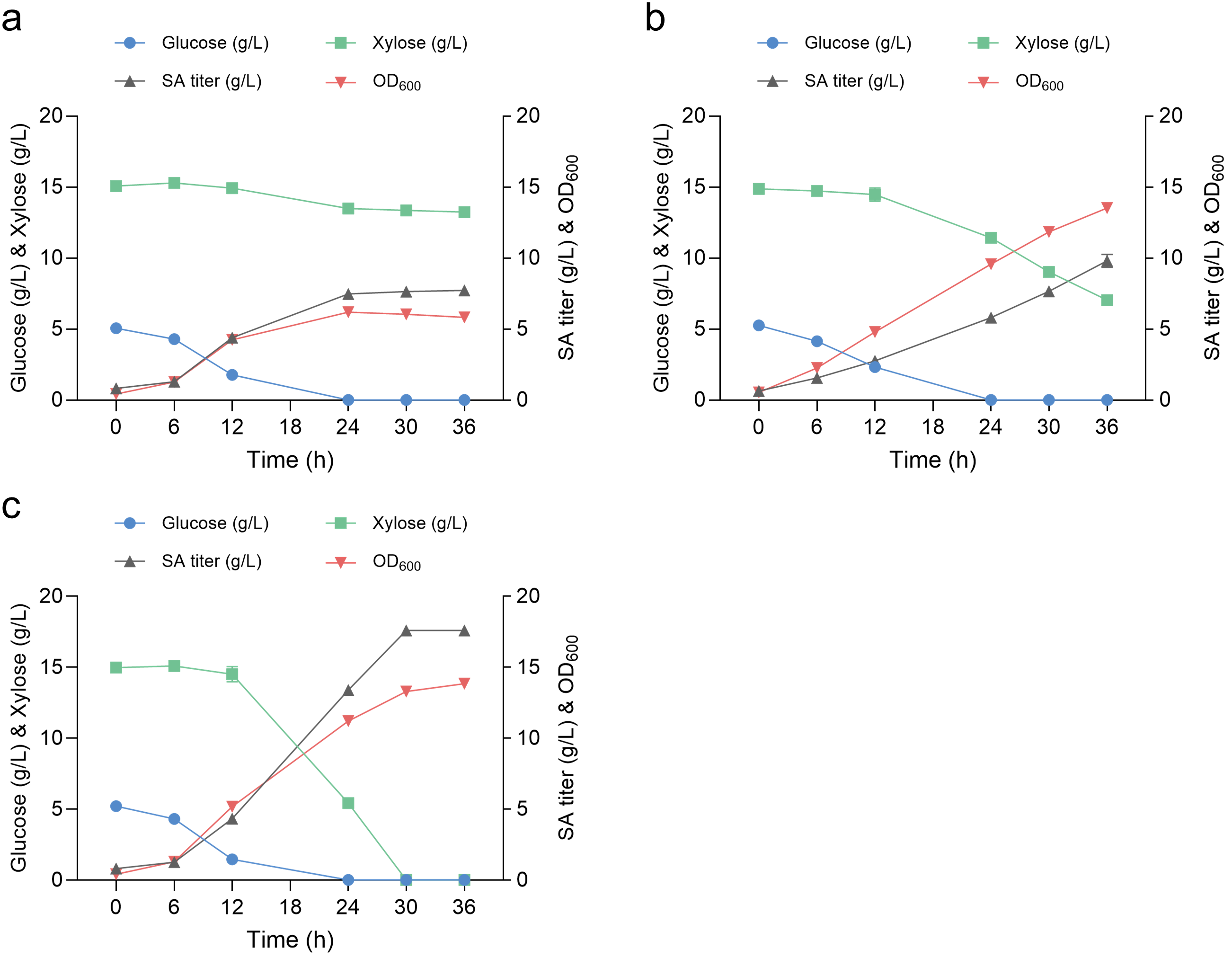


**Figure S13.** SA production from a glucose-xylose mixture by engineered *Y. lipolytica* strains. Shake-flask fermentation profiles of engineered strains Hi-SA0-X4 (a), EX413 (b) and Hi-SA0-G4 (c) in YPDX medium. Error bars represent mean ± s.d. (n = 3 biologically independent samples).

**Table S1.** Mutations identified in five evolved strains from Hi-SA0-X4.

**Table S2.** Information about shared mutations from five evolved strains.

| **Mutant type** | | **Genes** | **Name** | **Mutant site** | **Gene annotation** |
| --- | --- | --- | --- | --- | --- |
| SNV | YALI0D02101g | | Snf1^R78W^ | R78W | Snf1 Carbon catabolite-derepressing protein kinase |
| InDel | YALI0F00968g | | Scp1^delGTC^ | 2841-2843 delGTC | Scp1 Sterol-sensing domain of Sre1 cleavage-activation-domain-containing protein |
| SNV | YALI0E23001g | | Rho1^G156S^ | G156S | Rho1 Ras homolog gene family, member A |

**Table S3.** Comparison of glucose and xylose consumption rates of mutants within 48 h.

| **Strains** | **Xylose consumption rate (g/L/h)** | **Glucose consumption rate (g/L/h)** |
| --- | --- | --- |
| Po1fX-Snf1 | 0.30 | 0.46 |
| Po1fX-Snf1^R78W^ | 0.31 | 0.43 |
| Po1fX-Scp1 | 0.27 | 0.42 |
| Po1fX-Scp1^delGTC^ | 0.34 | 0.39 |
| Po1fX-Rho1 | 0.32 | 0.42 |
| Po1fX-Rho1^G156S^ | 0.29 | 0.43 |

**Table S4.** Transcriptome analysis of Snf1^R78W^, Scp1^delGTC^, and Rho1^G156S^ mutant strains.

**Table S5.** Transcriptome analysis of the evolved strain EX413 and the unevolved strain Hi-SA0-X4.

**Table S6.** Bio-based SA production from xylose and lignocellulosic hydrolysates by different microbial cell factories.

| **Strains** | **Substrate** | **Medium** | **pH control mode** | **Fermentation mode** | **Glucose consumed (g/L)** | **Xylose consumed (g/L)** | | **Succinic acid** | | | **Reference** |
| --- | --- | --- | --- | --- | --- | --- | --- | --- | --- | --- | --- |
|  |  |  |  |  |  |  |  | **Titer (g/L)** | **Yield (g/L)** | **Productivity (g/L)** |  |
| *E. coli* KJ12201-14T | Xylose | AM1 | pH was maintained at 7.0 by a mixture solution of 6 M KOH and 3 M K_2_CO_3_ | Fed-batch fermentation | N/A | | 98.9 | 84.6 | 0.86 | 1.01 | [1] |
| *E. coli* FZ661T | Woody biomass | NBS | pH was maintained at 6.8 by NH_4_OH | Dual‑stage anaerobic fermentation | 60 g/L mixed sugars | | | 54.5 | N/A | 1.81 | [2] |
| *C. glutamicum*  K5 | Corn stover | CGXII | pH was controlled by 200 mM NaHCO_3_ and 30 g/L Mg_2_(OH)_2_CO_3_ | Two-stage fermentation | 67.68 | | 16.5 | 64.16 | N/A | 1.07 | [3] |
| *C. glutamicum* CGS5 | Corn stalk | CGXIIB | pH was controlled by 4MgCO_3_·Mg(OH)_2_·5H_2_O | Two-stage, aerobic culture of biomass and anaerobic batch fermentation | 71.0 | | 30.1 | 98.6 | 0.87 | 4.29 | [4] |
| *Actinobacillus succinogenes* 130Z | Corn stover | Defined medium | pH was maintained at 6.8 by 2.5 M Na_2_CO_3_ | Batch fermentation | N/A | | N/A | 42.8 | 0.74 | 1.27 | [5] |
| *Basfia succiniciproducens* CCUG 57335 | Corn stover | Base media | pH was maintained at 6.8 by 4 N NaOH | Batch fermentation | N/A | | N/A | 30 | 0.69 | 0.43 | [6] |
| *Y. lipolytica* PSA02004PP | Xylose | Minimal medium | Without pH control during fermentation | Fed-batch fermentation | N/A | | 150 | 22.3 | 0.15 | 0.14 | [7] |
|  | Sugarcane bagasse |  |  | Batch fermentation | N/A | | 37.8 | 5.6 | 0.13 | 0.09 |  |
| *Y. lipolytica* PSA02004 | Sugarcane bagasse | Defined medium | pH was maintained at 6.0 by 5 M NaOH | Free-cell batch fermentation | 47.3 | | 10.2 | 33.2 | 0.58 | 0.33 | [8] |
| *Y. lipolytica* Hi-SA2-YlGsh2 | Corncob | CM1 | Without pH control during fermentation | Fed-batch fermentation | N/A | | 6.2 | 45.34 | 0.71 | 1.42 | [9] |
| *Y. lipolytica* BDic5 | Corn stover | YP | pH was adjusted to 6.5 by 10 M NaOH every 24 h | Fed-batch fermentation | 190.52 | | 113.51 | 105.42 | 0.35 | 0.53 | [10] |
| *Y. lipolytica* Hi-SA0-G4 | Xylose | CM1X | pH was maintained at 3.5 by NH_4_OH | Fed-batch fermentation | N/A | | 142.94 | 85.48 | 0.60 | 1.24 | This study |
|  | Corn stover | CM1H |  |  | 76.83 | | 50.30 | 83.78 | 0.66 | 1.21 | This study |

N/A: Not available

**Table S7.** Yeast strains used in this study.

| **Strains** | **Descriptions** | **Sources** |
| --- | --- | --- |
| Hi-SA0 | *MatA*, *xpr2-322*, *axp-2*, *leu2-270*, *ura3-302*, *ΔSdh5*::*loxP, ΔAch1*::*loxP*, *YlPyc*, *TbFrd*, *EcFum*, *YlMdh1*, *Pgl1^G75S^*, *mTbFrd*, *YlMdh2*, *SpMae1* | [11] |
| Hi-SA0-X1 | Hi-SA0 ::*XR* (YALI0D07634g), *XDH* (YALI0E12463g), and *XK* (YALI0F10923g) | This study |
| Hi-SA0-X2 | Hi-SA0 ::*XR*, *XDH*, and *XK* | This study |
| Hi-SA0-X4 | Hi-SA0 ::*XR*, *XDH*, and *XK* | This study |
| EX103 | Evolved strain derived from Hi-SA0-X4 | This study |
| EX106 | Evolved strain derived from Hi-SA0-X4 | This study |
| EX205 | Evolved strain derived from Hi-SA0-X4 | This study |
| EX209 | Evolved strain derived from Hi-SA0-X4 | This study |
| EX413 | Evolved strain derived from Hi-SA0-X4 | This study |
| EX422 | Evolved strain derived from Hi-SA0-X4 | This study |
| EX425 | Evolved strain derived from Hi-SA0-X4 | This study |
| EX426 | Evolved strain derived from Hi-SA0-X4 | This study |
| EX427 | Evolved strain derived from Hi-SA0-X4 | This study |
| EX431 | Evolved strain derived from Hi-SA0-X4 | This study |
| EX436 | Evolved strain derived from Hi-SA0-X4 | This study |
| EX437 | Evolved strain derived from Hi-SA0-X4 | This study |
| EX438 | Evolved strain derived from Hi-SA0-X4 | This study |
| EX439 | Evolved strain derived from Hi-SA0-X4 | This study |
| Hi-SA0-A1 | Hi-SA0-X4 ::*XR* | This study |
| Hi-SA0-B1 | Hi-SA0-X4 ::*XDH* | This study |
| Hi-SA0-C1 | Hi-SA0-X4 ::*XK* | This study |
| Hi-SA0-D1 | Hi-SA0-X4 ::*XR* and *XDH* | This study |
| Hi-SA0-E1 | Hi-SA0-X4 ::*XR* and *XK* | This study |
| Hi-SA0-F1 | Hi-SA0-X4 ::*XDH* and *XK* | This study |
| Hi-SA0-G4 | Hi-SA0-X4 ::*XR*, *XDH*, and *XK* | This study |
| Po1f | *MatA*, *leu2-270*, *ura3-302*, *xpr2-322*, *axp-2* | INRA |
| Po1fX | Po1f ::*XR*, *XDH*, and *XK* | This study |
| Po1fX-Snf1 | Po1fX Δ*Snf1* ::*Snf1* | This study |
| Po1fX-Snf1^R78W^ | Po1fX Δ*Snf1* ::*Snf1^R78W^* | This study |
| Po1fX-Scp1 | Po1fX Δ*Scp1* ::*Scp1* | This study |
| Po1fX-Scp1^delGTC^ | Po1fX Δ*Scp1* ::*Scp1^delGTC^* | This study |
| Po1fX-Rho1 | Po1fX Δ*Rho1* ::*Rho1* | This study |
| Po1fX-Rho1^G156S^ | Po1fX Δ*Rho1* ::*Rho1^G156S^* | This study |
| PGC91-rTE1-1 | Evolved strain derived from PGC91-rT strain and with the mutation of Pgll^G75S^ | [11] |
| PGC91-rTEX | PGC91-rTE1-1 ::*XR*, *XDH*, and *XK* | This study |
| PGC91-rTEX ΔPEX10 | PGC91-rTEX Δ*PEX10* (YALI0C01023g) | This study |
| PGC91-rTEX-mFRD | PGC91-rTEX ::*mTbFrd* (mitochondrial fumarate reductase) | This study |
| PGC91-rTEX-mFRD ΔPEX10 | PGC91-rTEX Δ*PEX10* ::*mTbFrd* | This study |

INRA: National Institute for Agricultural Research

**Table S8.** The culture medium used for strain cultivation in this study.

| **Name** | **Components** | **Experiments** |
| --- | --- | --- |
| LB | 5 g/L yeast extract (Oxoid), 10 g/L tryptone (Oxoid) and 10 g/L NaCl (Sinopharm); 20g/L agar powder (Solarbio) was added as needed for preparation of plates; 100 μg/mL ampicillin (Solarbio) was added as needed | Plasmid construction |
| YPD | 10 g/L yeast extract, 20 g/L tryptone and 20-60 g/L glucose (Sinopharm); 20g/L agar powder was added as needed for preparation of plates | Activation of culture; Fermentation |
| YPX | YP supplemented with 20-60 g/L xylose (Sinopharm); 20g/L agar powder was added as needed for preparation of plates | Fermentation; Screening of yeast transformants |
| YPDX | YP supplemented with an appropriate mixture of glucose and xylose | Fermentation; Adaptive evolution |
| YPDA | YPD supplemented with appropriate concentration of acrylic acid | β-Oxidation inhibition test on glucose |
| YPDC | YPD supplemented with appropriate concentration of cerulenin | Fatty acid synthesis inhibition test on glucose |
| YPXA | YPX supplemented with appropriate concentration of acrylic acid | β-Oxidation inhibition test on xylose |
| YPXC | YPX supplemented with appropriate concentration of cerulenin | Fatty acid synthesis inhibition test on xylose |
| CM1X | 0.8 g/L Na_2_HPO_4_·12H_2_O (Sinopharm), 3.6 g/L KH_2_PO_4_ (Sinopharm), 1.2 g/L MgSO_4_·7H_2_O (Sinopharm), 2.8 g/L (NH_4_)_2_SO_4_ (Sinopharm), 6.0 g/L corn steep powder (Solarbio) and 60 g/L xylose | Fed-batch fermentation |
| CM1H | CM1 supplemented with 16% (v/v) lignocellulosic hydrolysate | Fed-batch fermentation |
| SD-Ura | 26.7 g/L minimal synthetic defined (SD) bases (Takara) and 0.78 g/L Ura dropout (DO) supplement (Takara); 20g/L agar powder was added as needed for preparation of plates | Screening of yeast transformants |
| SD-Leu | 26.7 g/L minimal SD bases (Takara) and 0.70 g/L Leu DO supplement (Takara); 20g/L agar powder was added as needed for preparation of plates | Screening of yeast transformants |
| YNBD | 6.7 g/L yeast nitrogen base (YNB, Solarbio) and 40 g/L glucose | Growth test on glucose |
| YNBX | 6.7 g/L YNB and 40 g/L xylose | Growth test on xylose |

**Table S9.** Plasmids used in this study.

| **Plasmids** | **Descriptions** | **Sources** |
| --- | --- | --- |
| pUC19 | Standard *E. coli* vector with a multiple cloning site (MCS) for DNA cloning | Our lab |
| pUC19-rDNA-Ura-XR-XDH-XK | pUC19 harboring *Ura3* marker and expression cassettes of *XR*, *XDH*, and *XK* | This study |
| pUC19-rDNA-Leu-XR-XDH-XK | pUC19 harboring *Leu2* marker and expression cassettes of *XR*, *XDH*, and *XK* | This study |
| pUC19-rDNA-Leu-XR | pUC19 harboring *Leu2* marker and expression cassette of *XR* | This study |
| pUC19-rDNA-Leu-XDH | pUC19 harboring *Leu2* marker and expression cassette of *XDH* | This study |
| pUC19-rDNA-Leu-XK | pUC19 harboring *Leu2* marker and expression cassette of *XK* | This study |
| pUC19-rDNA-Leu-XR-XDH | pUC19 harboring *Leu2* marker and expression cassettes of *XR* and *XDH* | This study |
| pUC19-rDNA-Leu-XR-XK | pUC19 harboring *Leu2* marker and expression cassettes of *XR* and *XK* | This study |
| pUC19-rDNA-Leu-XDH-XK | pUC19 harboring *Leu2* marker and expression cassettes of *XDH* and *XK* | This study |
| pUC19-rDNA-Ura-Snf1 | pUC19 harboring *Ura3* marker and expression cassette of *Snf1* | This study |
| pUC19-rDNA-Ura-Snf1^R78W^ | pUC19 harboring *Ura3* marker and expression cassette of *Snf1^R78W^* | This study |
| pUC19-rDNA-Ura-Scp1 | pUC19 harboring *Ura3* marker and expression cassette of *Scp1* | This study |
| pUC19-rDNA-Ura-Scp1^delGTC^ | pUC19 harboring *Ura3* marker and expression cassette of *Scp1^delGTC^* | This study |
| pUC19-rDNA-Ura-Rho1 | pUC19 harboring *Ura3* marker and expression cassette of *Rho1* | This study |
| pUC19-rDNA-Ura-Rho1^G156S^ | pUC19 harboring *Ura3* marker and expression cassette of *Rho1^G156S^* | This study |
| pCAS1yl-gSnf1 | pCAS1yl harboring Snf1 gRNA module and Cas9 expression cassette | This study |
| pCAS1yl-gScp1 | pCAS1yl harboring Scp1 gRNA module and Cas9 expression cassette | This study |
| pCAS1yl-gRho1 | pCAS1yl harboring Rho1 gRNA module and Cas9 expression cassette | This study |
| pCAS1yl-gPEX10 | pCAS1yl harboring PEX10 gRNA module and Cas9 expression cassette | This study |
| pKi-1-mTbFrd | Integrative vector with the *Leu2* marker and MTS-TbFrd expression cassette | [11] |
| pClone007-XR | A standard plasmid harboring *XR* gene for qPCR standard curve construction | This study |
| pClone007-XDH | A standard plasmid harboring *XDH* gene for qPCR standard curve construction | This study |
| pClone007-XK | A standard plasmid harboring *XK* gene for qPCR standard curve construction | This study |
| pClone007-Actin | A standard plasmid harboring *Actin* gene for qPCR standard curve construction | This study |

**Table S10.** Primer sequences used in this study.

| **Primers** | **Primer sequences (5’-3’)** | **Functions** |
| --- | --- | --- |
| XR-F | AATTAAACACACATCAACAGATGTCCTTCAAGCTCGCCTC | Amplification of *XR* |
| XR-R | GGACAGGCCATGGAGGTACTTATTAGGCGAAAATGGGAAGGTTAGC | Amplification of *XR* |
| XDH-F | CTTTTTGCAGTACTAACCGCAGATGTCTTCTAACCCGTCATTTGTTCTTC | Amplification of *XDH* |
| XDH-R | CGTGACATAACTAATTACATGACTACTCCTCCTCGGGACCGT | Amplification of *XDH* |
| XK-F | ACAAGACATATCTACAGCAGATCTATGTATCTCGGACTGGATCTTTCGAC | Amplification of *XK* |
| XK-R | ATTTTGCTAAACAAACTGCAGATCTTTATTTCTCCAGGCAGGCGTTTTCG | Amplification of *XK* |
| Snf1-F | CCCGGTACGCGATCGCGTTTTGAAGCGGGAAATCAAGATTGAGAG | Amplification of *Snf1* and *Snf1^R78W^* expression cassettes |
| Snf1-R | GCGCTTGGTTGAATTTGTTTCCACTTTGAGAGTATCATCGTCAAGG | Amplification of *Snf1* and *Snf1^R78W^* expression cassettes |
| Scp1-F | CCCGGTACGCGATCGCGTTTCCAGATATCCTGCACACCACTG | Amplification of *Scp1* and *Scp1^delGTC^* expression cassettes |
| Scp1-R | CCGCGCTTGGTTGAATTTGTTTAGTCCGAGTGTATTTAATGATTAAAATTGTCTG | Amplification of *Scp1* and *Scp1^delGTC^* expression cassettes |
| Rho1-F | CCCGGTACGCGATCGCGTTTACCGATGACAAGACCATTACCTGG | Amplification of *Rho1* and *Rho1^G156S^* expression cassettes |
| Rho1-R | CCGCGCTTGGTTGAATTTGTTTACAATATAACTAATTGTTGTATGAATGCAAGTACGTG | Amplification of *Rho1* and *Rho1^G156S^* expression cassettes |
| gSnf1-F | TCTATGGCAGTACTCGACTGGTTTTAGAGCTAGAAATAGCAAGTTAAAATAAGGCTAGT | Construction of pCAS1yl-gSnf1 |
| gSnf1-R | CAGTCGAGTACTGCCATAGATGCGCCGACCCGGAATC | Construction of pCAS1yl-gSnf1 |
| gScp1-F | TGTCAAACATCACATCGGTGGTTTTAGAGCTAGAAATAGCAAGTTAAAATAAGGCTAGT | Construction of pCAS1yl-gScp1 |
| gScp1-R | CACCGATGTGATGTTTGACATGCGCCGACCCGGAATC | Construction of pCAS1yl-gScp1 |
| gRho1-F | TCTGCTGGTCGGTTGCAAGGGTTTTAGAGCTAGAAATAGCAAGTTAAAATAAGGCTAGT | Construction of pCAS1yl-gRho1 |
| gRho1-R | CCTTGCAACCGACCAGCAGATGCGCCGACCCGGAATC | Construction of pCAS1yl-gRho1 |
| gPEX10-F | GAGACCGAACAGCTCTACCGGTTTTAGAGCTAGAAATAGCAAGTTAAAATAAGGCTAGT | Construction of pCAS1yl-gPEX10 |
| gPEX10-R | CGGTAGAGCTGTTCGGTCTCTGCGCCGACCCGGAATC | Construction of pCAS1yl-gPEX10 |
| qP-XR-F | TGTGGAGATTGGCATGGACC | Detection of the *XR* gene |
| qP-XR-R | GCTCGACGTTGTTGGACTTG | Detection of the *XR* gene |
| qP-XDH-F | AGTTCGGTGCCACTCATACG | Detection of the *XDH* gene |
| qP-XDH-R | CACATTGGGCGAGTCAGAGA | Detection of the *XDH* gene |
| qP-XK-F | GCCTCGGTCAACACTGCTAT | Detection of the *XK* gene |
| qP-XK-R | TTCGTGAACCTGTACCTGGC | Detection of the *XK* gene |
| qP-Actin-F | ATGGCCGAGTCATCACCATC | Detection of the *Actin* gene |
| qP-Actin-R | TCTCCTTGTGCATTCGCTCG | Detection of the *Actin* gene |

**Table S11.** Changes in glucose and xylose concentration gradient during the ALE process.

| **Time (d)** | **Concentration gradient** | |
| --- | --- | --- |
|  | **Glucose (g/L)** | **Xylose (g/L)** |
| 0 | 32 | 8 |
| 7 | 16 | 24 |
| 19 | 8 | 32 |
| 31 | 4 | 36 |
| 66 | 2 | 38 |
| 116 | 1 | 38 |
| 146 | 0 | 40 |
| 231 | 0 | 40 |

**Supplementary References**

[1] P. Khunnonkwao, S. S. Jantama, S. Kanchanatawee, K. Jantama, *Appl Microbiol Biotechnol* **2018**, *102* (1), 127.

[2] F. Zhu, C. Wang, K. Y. San, G. N. Bennett, *J Ind Microbiol Biotechnol* **2020**, *47* (2), 223.

[3] K. Li, C. Li, X. Q. Zhao, C. G. Liu, F. W. Bai, *Bioresour Technol* **2023**, *378*, 128991.

[4] Y. Mao, G. Li, Z. Chang, R. Tao, Z. Cui, Z. Wang, Y. J. Tang, T. Chen, X. Zhao, *Biotechnol Biofuels* **2018**, *11*, 95.

[5] D. Salvachua, A. Mohagheghi, H. Smith, M. F. A. Bradfield, W. Nicol, B. A. Black, M. J. Biddy, N. Dowe, G. T. Beckham, *Biotechnol Biofuels* **2016**, *9*, 28.

[6] D. Salvachua, H. Smith, P. C. St John, A. Mohagheghi, D. J. Peterson, B. A. Black, N. Dowe, G. T. Beckham, *Bioresour Technol* **2016**, *214*, 558.

[7] A. A. Prabhu, R. Ledesma-Amaro, C. S. K. Lin, F. Coulon, V. K. Thakur, V. Kumar, *Biotechnol Biofuels* **2020**, *13*, 113.

[8] K. L. Ong, C. Li, X. Li, Y. Zhang, J. Xu, C. S. K. Lin, *Biochemical Engineering Journal* **2019**, *148*, 108.

[9] Y. Zhong, J. Gu, C. Shang, J. Deng, Y. Liu, Z. Cui, X. Lu, Q. Qi, *Bioresour Technol* **2024**, *408*, 131166.

[10] M. Ge, Y. Sha, M. Lu, Y. Zhang, Z. Xu, S. Chen, Y. Ding, M. Jin, *Green Chem* **2025**, *27* (2), 450.

[11] Z. Cui, Y. Zhong, Z. Sun, Z. Jiang, J. Deng, Q. Wang, J. Nielsen, J. Hou, Q. Qi, *Nat Commun* **2023**, *14* (1), 8480.
